# Interactions of *BDNF* Val66Met Polymorphism and Menstrual Pain on Brain Complexity

**DOI:** 10.3389/fnins.2018.00826

**Published:** 2018-11-20

**Authors:** Intan Low, Po-Chih Kuo, Cheng-Lin Tsai, Yu-Hsiang Liu, Ming-Wei Lin, Hsiang-Tai Chao, Yong-Sheng Chen, Jen-Chuen Hsieh, Li-Fen Chen

**Affiliations:** ^1^Institute of Brain Science, National Yang-Ming University, Taipei, Taiwan; ^2^Integrated Brain Research Unit, Department of Medical Research, Taipei Veterans General Hospital, Taipei, Taiwan; ^3^Institute of Statistical Science, Academia Sinica, Taipei, Taiwan; ^4^Institute of Biomedical Informatics, National Yang-Ming University, Taipei, Taiwan; ^5^Institute of Public Health, National Yang-Ming University, Taipei, Taiwan; ^6^Department of Obstetrics and Gynecology, Taipei Veterans General Hospital, Taipei, Taiwan; ^7^Department of Computer Science, National Chiao Tung University, Hsinchu, Taiwan; ^8^Center for Emergent Functional Matter Science, National Chiao Tung University, Hsinchu, Taiwan; ^9^Brain Research Center, National Yang-Ming University, Taipei, Taiwan

**Keywords:** *BDNF* Val66Met polymorphism, primary dysmenorrhea, brain complexity, multiscale entropy, magnetoencephalography, chronic pain

## Abstract

The irregularity and uncertainty of neurophysiologic signals across different time scales can be regarded as neural complexity, which is related to the adaptability of the nervous system and the information processing between neurons. We recently reported general loss of brain complexity, as measured by multiscale sample entropy (MSE), at pain-related regions in females with primary dysmenorrhea (PDM). However, it is unclear whether this loss of brain complexity is associated with inter-subject genetic variations. Brain-derived neurotrophic factor (BDNF) is a widely expressed neurotrophin in the brain and is crucial to neural plasticity. The *BDNF* Val66Met single-nucleotide polymorphism (SNP) is associated with mood, stress, and pain conditions. Therefore, we aimed to examine the interactions of *BDNF* Val66Met polymorphism and long-term menstrual pain experience on brain complexity. We genotyped *BDNF* Val66Met SNP in 80 PDM females (20 Val/Val, 31 Val/Met, 29 Met/Met) and 76 healthy female controls (25 Val/Val, 36 Val/Met, 15 Met/Met). MSE analysis was applied to neural source activity estimated from resting-state magnetoencephalography (MEG) signals during pain-free state. We found that brain complexity alterations were associated with the interactions of *BDNF* Val66Met polymorphism and menstrual pain experience. In healthy female controls, Met carriers (Val/Met and Met/Met) demonstrated lower brain complexity than Val/Val homozygotes in extensive brain regions, suggesting a possible protective role of Val/Val homozygosity in brain complexity. However, after experiencing long-term menstrual pain, the complexity differences between different genotypes in healthy controls were greatly diminished in PDM females, especially in the limbic system, including the hippocampus and amygdala. Our results suggest that pain experience preponderantly affects the effect of *BDNF* Val66Met polymorphism on brain complexity. The results of the present study also highlight the potential utilization of resting-state brain complexity for the development of new therapeutic strategies in patients with chronic pain.

## Introduction

Chronic pain can be considered as “pain that persists for a given length of time,” where the length of time is determined by common medical experience (Merskey and Bogduk, 2002), and have pronounced female predominance (Mogil, 2012). Primary dysmenorrhea (PDM) indicates “pain with menstruation not associated with a well-defined pathology”^1^ and is classified as chronic pelvic pain syndrome (IASP, 2011). It has a prevalence of 45–95% among reproductive age females (Berkley, 2013; Iacovides et al., 2015). Most importantly, dysmenorrhea early in life often co-occurs with many chronic pain syndromes later in life, linking its susceptibility to the development of chronic pain conditions (Berkley, 2013) and marking the assaults of central sensitization on dysfunctional pain modulatory system (Nijs et al., 2015).

Long-term PDM serves as a genuine model to study clinical pain with its natural cyclic painful (menstruation) and pain-free (periovulatory) states (Wei et al., 2016a). Structural and functional brain alterations are reported in PDM females (PDMs), including hypotrophic and hypertrophic changes in gray matter volume (Tu et al., 2010, 2013; Liu et al., 2016), white matter microstructural alterations (Liu J. et al., 2017), maladaptive descending pain modulatory system (Wei et al., 2016a), shift of functional connectivity between resting-state networks (Wu et al., 2016; Liu P. et al., 2017), increased theta activity (Lee et al., 2017), and loss of brain complexity (Low et al., 2017) in brain regions related to sensory, affective, and cognitive dimensions of pain. Notably, genetic polymorphisms have been implicated to contribute to inter-subject variations in susceptibility to menstrual pain (Lee et al., 2014; Wei et al., 2016b, 2017), inviting more studies of neuroimaging genetics in PDM.

Brain-derived neurotrophic factor (BDNF) is the most expressed neurotrophin in the brain, especially in the cerebral cortex and hippocampus (Binder and Scharfman, 2004; Tapia-Arancibia et al., 2004). BDNF has pleiotropic roles in the central nervous system, including neurogenesis, neuronal growth, maturation, survival, synaptic plasticity, and microarchitectural integrity (Park and Poo, 2013; Bathina and Das, 2015). It is a driving force behind neural plasticity and protects neuronal cells upon adverse circumstances (Bathina and Das, 2015), such as stress or pain. BDNF is considered as a pain modulator given its participation in activity-dependent synaptic plasticity within the pain modulatory circuitry (Merighi et al., 2008; Haas et al., 2010; Caumo et al., 2016; Generaal et al., 2016).

Human *BDNF* gene at chromosome 11p14.1 displays a variety of polymorphisms. The replacement of Valine (Val) by Methionine (Met) at codon 66, namely *BDNF* Val66Met single-nucleotide polymorphism (rs6265), is considered to disrupt normal trafficking of BDNF and consequently reduces activity-dependent secretion of BDNF and BDNF activity in Met carriers (Egan et al., 2003). Animal studies reported that spike-timing-dependent plasticity in the pyramidal neurons of the infralimbic medial prefrontal cortex was absent in *BDNF* Met/Met mice, suggesting that *BDNF* Val66Met polymorphism strongly affect synaptic transmission (Pattwell et al., 2012). The *BDNF* Met allele has been reported to associate with deleterious effects on brain, such as smaller regional brain volumes (Pezawas et al., 2004), higher vulnerability in white matter structural connectivity (Park et al., 2017), and potentially greater susceptibility to various neurological and mood disorders (Notaras et al., 2015). Studies of the effects of *BDNF* Val66Met polymorphism on pain also predominantly report impaired pain modulation or augmented pain responses in Met carriers, including migraine (Cai et al., 2017), chronic musculoskeletal pain (Generaal et al., 2016), chronic abdominal pain (Reddy et al., 2014), electrical stimulation for trigeminal pain (Di Lorenzo et al., 2012), intracutaneous pain in chronic low back pain patients (Vossen et al., 2010), and esophageal visceral pain (Vasant et al., 2011). However, our understanding of the role of *BDNF* genetic variants in recurrent menstrual pain is still limited.

Previous *BDNF* Val66Met polymorphism studies in PDM observed a significant main effect of *BDNF* genotype on anxiety level in PDM group, in which Met/Met PDMs scored higher in anxiety compared with Val-carrier PDMs during menstrual phase (Lee et al., 2014). The authors suggested that *BDNF* Val66Met polymorphism is modestly associated with the supraspinal modulation of menstrual pain-laden emotional processing. On the other hand, resting-state functional connectivity study in PDM (Wei et al., 2016b) revealed that Val/Val PDMs engaged functional connectivity between pain modulatory region and sensory regions, suggesting adaptive pain modulation, while Met/Met PDMs rigidly engaged functional connectivity between pain modulatory region and limbic structures, implying maladaptive pain modulation underlying pain chronicity. Together, *BDNF* Val66Met polymorphism might affect spontaneous low-frequency BOLD signal oscillations differently in individuals with or without long-term menstrual pain experience.

The irregularity and unpredictability (uncertainty) of a system's output signal across varying temporal scales can be regarded as the system's complexity (Costa et al., 2002). Neural complexity, the complexity of the nervous system, could represent the capacity or dynamical range of information processing in the brain, the richness of information available in the nervous system, or adaptability or resilience of the nervous system (Tononi et al., 1994; Nakagawa et al., 2013; McDonough and Nashiro, 2014; Wang et al., 2018). Neural complexity might reflect the brain's tendency to wander (itinerancy) among all alternative states of neuronal transients, and as a characterization of the “flexibility of rapid transitions” (Friston, 2000, 2001; Friston et al., 2012; Wang et al., 2018). Loss of complexity is often reported in neuropsychiatric diseased and aged groups; increased complexity has been seen in recovery conditions and healthy groups (Yang and Tsai, 2013; Hager et al., 2017).

Complexity is non-linear and complex to define but is often computationally quantified with entropy measurements. Sample entropy, proposed by Richman and Moorman (Richman and Moorman, 2000), is a well-defined index of complexity and has been applied to brain activity (Yao et al., 2013; Wang et al., 2014, 2017; Lebedev et al., 2016; Li et al., 2016; Zhou et al., 2016; Jia et al., 2017; Nelson et al., 2017; Chang et al., 2018; Song et al., 2018). It is noteworthy that multiscale entropy (MSE) analysis (Costa et al., 2002, 2005; Yang and Tsai, 2013; Courtiol et al., 2016) calculates a series of sample entropy over multiple time scales, which captures the temporal complexity characteristcs of time-series neural signals from microscopic to macroscopic aspects. Recently, MSE analysis has also been applied to brain signals (Heisz and McIntosh, 2013; Yang et al., 2013; Courtiol et al., 2016), pain (Sitges et al., 2010; Valencia et al., 2016; Liu Q. et al., 2017), and PDM studies (Kuo et al., 2017; Low et al., 2017). By applying MSE analysis on resting-state magnetoencephalography (MEG) signals acquired from PDMs during pain-free state, we observed a general loss of regional complexity in PDMs at brain regions related to chronic pain, including the limbic circuitry, default mode network, sensorimotor network, and salience network (Low et al., 2017). Our findings implicated the assaults of long-term menstrual pain on brain complexity and adaptability. However, it is unclear whether this loss of brain complexity in PDMs is associated with genetic variations.

Long-term menstrual pain is a chronic stressor to PDMs that might affect the secretion levels of BDNF and subsequent BDNF functions in an activity-dependent manner. Given the loss of brain complexity in PDMs and the effects of *BDNF* Val66Met polymorphism on mood and resting-state functional connectivity in PDMs, we aimed to examine the interactions of *BDNF* Val66Met polymorphism and long-term menstrual pain experience on brain complexity. We hypothesized that there might be genotype-specific complexity differences in healthy controls, and such complexity differences might be affected by long-term menstrual pain especially in pain- and emotion-related brain circuits.

## Methods

### Participants

The participants were a subset of the participants from our multimodal imaging genetics (magnetic resonance imaging and magnetoencephalography) and behavioral studies of PDM at Taipei Veterans General Hospital in Taiwan (Lee et al., 2014; Wei et al., 2016a; Wu et al., 2016; Low et al., 2017) who were eligible for neuroimaging studies. Written informed consent form and psychological inventories were approved by the ethics committee of Institutional Review Board of Taipei Veterans General Hospital, Taiwan. Before the study, all participants who were assessed for eligibility signed the written informed consent form. Studies were conducted in accordance with the Declaration of Helsinki.

The inclusion criteria for PDMs were (1) 20 to 30 years old Taiwanese (Asian) females; (2) 27 to 32 days of regular menstrual cycle; (3) right-handedness assessed by the Edinburgh Handedness Inventory; (4) menstrual pain history longer than half year; (5) averaged menstrual pain rating within the last 6 months of experiment was higher than four out of ten using verbal numerical rating scale (0 = no pain, 10 = worst imaginable pain); (6) no pelvic pathologies examined using pelvic ultrasonography and diagnosed as PDM by gynecologist. We excluded volunteers with (1) organic pelvic diseases; (2) pituitary gland pathologies; (3) history of neurological or psychiatric disorders; (4) history of brain surgery or trauma; (5) history or immediate plans for pregnancy or childbirth; (6) history of using medications or supplements of hormonal therapy including oral contraceptives, central-acting medication, or Chinese herbal medicine within the last 6 months of experiment; (7) claustrophobia; (8) contraindications to magnetic resonance imaging. Also, no painkillers were used 24 h before the experiment. Healthy female controls (CONs) had the same inclusion and exclusion criteria except they had no lower abdominal pain during the menstrual period.

### Genotyping

Whole blood was collected during the inception stage and stored in 4 mL EDTA tubes at 4°C refrigerator. DNA was extracted using the Puregene kit following the manufacturer's guidelines (Gentra Systems, Inc., Minneapolis, MN, USA). Genotyping was conducted using commercial TaqMan single-nucleotide polymorphism assays (Applied Biosystems, Inc., Foster City, CA, USA). The polymerase chain reaction amplification protocol was as follows: 10 μL; 50°C (2 min), 95°C (10 min), 40 cycles of 92°C (15 s), and 60°C (1 min). Fluorescence measurements were done using the ABI HT7900 (Applied Biosystems, Inc.). Allele calling was performed by the SDS 2.2 software package (Applied Biosystems, Inc.). Two independent technicians blinded to the participants' personal information assigned the genotypes.

### Demographic data, pain experiences, and psychological characteristics

Demographic data included age, body mass index (BMI), and handedness. Menstrual features included age at menarche, years of menstruating, and averaged menstrual cycle length. All participants completed the Chinese version of Basic Personality Inventory (BPI; Wu et al., 1999) to assess their personality traits. There are several scale clusters in the BPI, including the personal emotional adjustment scale cluster (depression, anxiety, and hypochondriasis scales), which is of particular interest of this study. The IQOLA SF-36 Taiwan Standard Version 1.0 (SF-36; Tseng et al., 2003) was used to assess long-term physical and mental quality of life. Since emotional perception and pain chronification can be exacerbated by anxiety and depression and both have been linked to PDM, anxious and depressive moods were investigated using Chinese versions of Spielberger State-trait Anxiety Inventory (STAI; Ma et al., 2013), Beck Depression Inventory (BDI-IA; Beck et al., 1979), and Beck Anxiety Inventory (BAI; Lin, 2000). We also studied pain catastrophizing cognitive style, which is the negative appraisal style of pain, using the Chinese version of Pain Catastrophizing Scale (PCS; Yap et al., 2008).

Menstrual pain experiences were evaluated only in PDMs using the Chinese version of McGill Pain Questionnaire (MPQ; Melzack, 1975, 1983) and verbal numerical rating scale (VNRS). MPQ classifies four categories of qualities of pain, including sensory, affective, evaluative, and miscellaneous. MPQ scores are calculated as the sum of the rank values of the words chosen, summing up to a pain rating index (PRI) for each category. The MPQ present pain index (PPI), based on a 0-to-5 intensity scale (0 = no pain, 1 = mild, 2 = discomforting, 3 = distressing, 4 = horrible, 5 = excruciating), was used as the indicator of menstrual pain intensity. VNRS is a verbal report of menstrual pain intensity rated from 0 to 10 (0 = not at all, 10 = the worst imaginable pain). PDMs recalled different aspects of their overall menstrual pain experiences over the last 6 months, yielding recalled MPQ scores and recalled pain scores.

### Data acquisition

#### Resting-state MEG signals acquisition

Three-minute eye-closed resting-state MEG signals were recorded using a whole-head 306-channel neuromagnetometer (Vectorview, Elekta Neuromag, Helsinki, Finland) comprising 102 triple sensors (two orthogonal planar gradiometers and one magnetometer at each triple-set) at Taipei General Veterans Hospital, Taiwan. Electrooculography (EOG) was recorded using two vertical and two horizontal electrodes to be used for rejection of epochs coinciding with blinks and excessive eye movements with an amplitude cut-off of 600 μV. Locations of three anatomical landmarks (nasion and two bilateral pre-auricular points) were identified with a three-dimensional digitizer (Isotrak 3S10002, Polhemus Navigation Sciences, Colchester, Vermont USA) to align MEG coordinate system with MRI coordinate system. Four head position indicator (HPI) coils were used to trace the position of subject's head in the MEG system. The online sampling rate was 1,000 Hz, and online bandpass filter was between 0.03 and 330 Hz with a 60 Hz notch filter. Signals exceeding 6,000 fT/cm were rejected. MEG signals recorded from 204 planar gradiometers were further analyzed; MEG signals recorded from 102 magnetometers were excluded from further analyses due to their susceptibility to distant noises. Participants sat comfortably in a magnetically shielded room (Euroshield, Eura, Finland) with heads covered by the helmet and were instructed to relax, eliminate eye movements, and focus only on their breathing (Low et al., 2017).

#### Structural MRI images acquisition

T1-weighted brain images were acquired using a 3 Tesla magnetic resonance imaging (MRI) scanner (Magnetom Trio Tim, Siemens, Erlangen, Germany) at National Yang-Ming University, Taiwan with 12-channel head coil and standard three-dimensional magnetization-prepared rapid gradient-echo (3D MP-RAGE) sequence. The parameters were as follows (Low et al., 2017): TR = 2530 ms, TE = 3.03 ms, TI = 1,100 ms, flip angle = 7°, field-of-view (FOV) = 224 × 256 mm^2^, number of slices = 192, matrix size = 224 × 256, thickness = 1 mm.

### Brain region parcellation

A total of 90 cortical regions (45 regions in each hemisphere) were defined using the automated anatomical labeling (AAL) template (Tzourio-Mazoyer et al., 2002) with a spatial resolution of 1 × 1 × 1 mm provided in MRIcro freeware (Rorden and Brett, 2000). We first normalized the Montreal Neurological Institute (MNI) template to individual's MRI images using IBASPM (Individual Brain Atlases using Statistical Parametric Mapping; Alemán-Gómez et al., 2006). The AAL template was subsequently transformed into individual space using the estimated deformation field. Hence, parcellation of 90 brain regions in the individual brain was obtained, and voxel-wise source analyses (section Source Analyses) and MSE calculations (section Multiscale Sample Entropy) were carried out in each brain region. The purpose of this procedure was to avoid interpolation of functional activity and thus preserved the precision of MSE statistical analysis in individual space. Finally, eight resting-state networks based on literature were discussed, including the limbic, default mode, salience, sensorimotor, executive control, attention, visual processing, and auditory processing networks (Low et al., 2017).

### Source analyses

MEG signals from each participant were preprocessed before voxel-wise source reconstruction. The signals were segmented into non-overlapping epochs of 8 s (Low et al., 2017). Artifact rejection threshold was set to 2,000 fT/cm, and EOG rejection threshold was set to 250 μV. Any epoch with amplitude larger than these thresholds was excluded, resulting in around 14 eight-second epochs remained. Signal space projected MEG signals were subsequently band-pass filtered within 0.5–90 Hz (Low et al., 2017) and with a notch filter of 55–65 Hz. Zero-mean adjustment was also applied.

After co-registering the coordinate systems between individual's MRI volume and MEG device, the source activity was estimated with an isotropic resolution of 4 mm. Maximum contrast beamformer (MCB) was used for brain source calculation (Chen Y. S. et al., 2006). To estimate source activity *y*_*v*_(*t*) at each location *v*, a spatial filter **w**_*v*_ was applied to the MEG recordings **m**(*t*):

(1) $$\begin{array}{r} y_{v}\left(t\right)={\text{w}}_{v}^{\text{T}}\text{m}\left(t\right). \end{array}$$

For each location, **w**_*v*_ was obtained by minimizing variance in the output signal *y*_*v*_(*t*) with the unit-gain constraint ${\text{w}}_{v}^{\text{T}}\;{\text{l}}_{v}=1$, where **l**_*v*_ is the lead field vector. The details of the calculation can be found in our recent report (Kuo et al., 2017). Source activity of all voxels in every brain region was used in the following analysis.

### Multiscale sample entropy

Multiscale sample entropy (MSE) was proposed to measure the complexity with multiple time scales (Costa et al., 2002) by applying the sample entropy (SE) method (Richman and Moorman, 2000) to different time scales. First, the coarse-grained time series ${{\text{z}}_{v}}^{\tau }=\left[\;{z_{v}}^{\tau }\left(1\right),\;{z_{v}}^{\tau }\left(2\right),\;\ldots ,\;{z_{v}}^{\tau }\left(N/\tau \right)\right]$ of the original time series **y**_*v*_ = [ *y*_*v*_(1), *y*_*v*_(2), …, *y*_*v*_(*N*)] with *N* sample points was obtained for each scale factor τ (τ was set from 1 to 100 in this study; Low et al., 2017):

(2) $$\begin{array}{r} {z_{v}}^{\tau }\left(k\right)=\;\frac{1}{\tau }\sum_{i=\left(k-1\right)\tau \;+\;1}^{k\times \tau }y_{v}\left(i\right),\;1\;\leq k\leq \;\frac{N}{\tau }\;. \end{array}$$

Then the SE method was applied to each ${{\text{z}}_{v}}^{\tau }$. In the SE algorithm, a set of vector **g**_*i*_ with *m* elements can be defined as follows (*m* = 2 in this study; Low et al., 2017):

(3) $$\begin{array}{r} {\text{g}}_{i}=\;\left[\;{z_{v}}^{\tau }\left(i\right),\;{z_{v}}^{\tau }\left(i+1\right)\right],where\;i=1,\ldots ,N-2. \end{array}$$

For each *i*, the absolute difference between **g**_*i*_ and **g**_*j*_ (1 ≤ *j* ≤ *N*−2 and *j* ≠ *i*) was calculated, and $c_{\tau }^{g}$, the number of the difference smaller than *r*, was determined. Here, the value *r* was set as 0.25 × standard deviation of the signal *y*_*v*_(*t*) (Low et al., 2017). Then, the extended vectors with *m*+1 elements were defined as follows:

(4) $$\begin{array}{r} {\text{h}}_{i}=\;\left[\;{z_{v}}^{\tau }\left(i\right),\;{z_{v}}^{\tau }\left(i+1\right),\;{z_{v}}^{\tau }\left(i+2\right)\right],\\ where\;i=1,\ldots ,N-3, \end{array}$$

and $c_{\tau }^{h}$ was determined by the same means. Finally, MSE for time scale τ can be calculated as:

(5) $$\begin{array}{r} MSE_{\tau }\;=\;-\ln\;\frac{c_{\tau }^{h}}{c_{\tau }^{g}}. \end{array}$$

**Figure 1:**
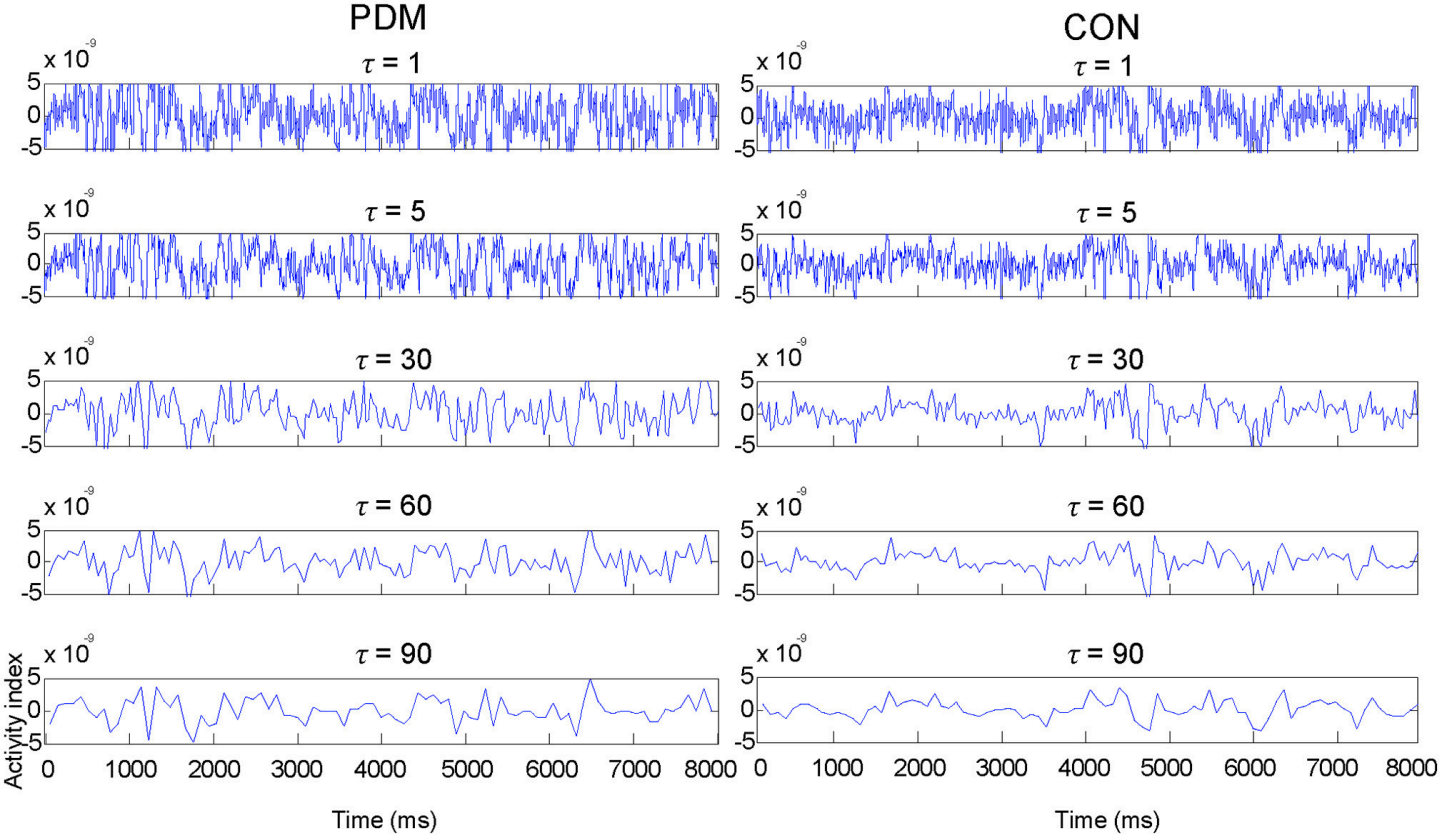
Examples of MEG signals at different representative time scale factors (τ) in one region (the left amygdala) from one representative subject in each group.

The MSE value of each voxel was calculated and then averaged within each region. Figure 1 illustrates examples of MEG signals at different representative time scale factors (τ) in one region (the left amygdala) from one representative subject in each group.

### Statistical analyses

There were two between-group factors in this study: group (PDM and CON) and *BDNF* Val66Met genotype (Val/Val, Val/Met, and Met/Met). Statistical analyses of genotypes, demographic data, psychological characteristics, and correlation analyses were performed in IBM SPSS Statistics. Statistical analyses of multiscale sample entropy were performed in Matlab.

#### *BDNF* genotype distributions and allele frequency

The Hardy-Weinberg equilibrium of the *BDNF* Val66Met genotype distributions and allele frequency were tested using *chi*-square tests of goodness-of-fit (*p* < 0.05). The associations between *BDNF* genotype and PDM were tested using *chi*-square tests of independence (*p* < 0.05) under SPSS binary logistic regression test, in which PDM (group) was treated as the dependent (outcome) variable, genotype as the categorical predictor variable, and Val/Val, Val/Met, or Val carriers as the reference groups.

#### Demographic data, pain experiences, and psychological characteristics

Descriptive and normality tests (Shapiro-Wilk test, *p* < 0.05, two-tailed) were first examined by group before any inferential statistical tests (Ghasemi and Zahediasl, 2012). As many of the demographic and psychological scores were not normally distributed, we used non-parametric inferential statistical tests. Group difference of each genotype was tested using Mann-Whitney *U* tests (*p* < 0.01, two-tailed). Genotype differences in each group were tested using Kruskal-Wallis *H* tests (*p* < 0.01, two-tailed) for continuous data and *chi*-square tests (*p* < 0.01) for categorical data. As there is no corresponding nonparametric two-way ANOVA test, significant group and genotype main effects and group × genotype interactions were tested using two-way ANOVAs (*p* < 0.01, two-tailed). *Post-hoc* pairwise comparisons were performed using Dunn-Bonferroni adjusted *p* < 0.01 (two-tailed).

#### Multiscale sample entropy

For the averaged MSE value in each brain region, the main effects and interactions of group and genotype were tested using two-way ANOVA (Bonferroni-adjusted *p* < 0.05, two-tailed). Due to possible errors induced by the above-mentioned normalization procedure (section Brain Region Parcellation), we excluded the brain regions that contained <10 voxels. We focused on testing the *post-hoc* planned pairwise comparisons between groups for each genotype and comparisons between genotypes for each group using permutation tests (iterations = 5000, *p* < 0.005, two-tailed). We also tested *post-hoc* planned comparisons using the more conservative Bonferroni-correction (Bonferroni-adjusted *p* < 0.05, two-tailed).

MSE of the brain regions that showed significant group differences in the same genotype group (Val/Val: PDM vs. CON; Val/Met: PDM vs. CON; Met/Met: PDM vs. CON) were termed as “pain-associated regional MSE.” MSE of the brain regions that showed significant genotype differences (Val/Val vs. Val/Met, Val/Val vs. Met/Met, Val/Met vs. Met/Met) in the same group were termed as “*BDNF*-associated regional MSE.” To examine the effect sizes of pain-associated regional MSE and *BDNF*-associated regional MSE, standardized effect sizes were calculated using Cohen's *d* (Cohen, 1988) with an online effect size calculator (Wilson and Lipsey, 2001).

#### Correlations between regional MSE and psychological characteristics

Correlations between PDMs' menstrual pain experiences/psychological characteristics and pain-associated or *BDNF*-associated regional MSE were examined using Spearman correlation analysis (*p* < 0.01, two-tailed).

## Results

### *BDNF* val66met genotype distributions and allele frequency

Genotype distributions of *BDNF* Val66Met (rs6265) in the CON group, PDM group, and all participants were in Hardy-Weinberg equilibrium (*p* > 0.05; Table S1). The number of participants in each genotype subgroup were as follows (Table 1): Val/Val CONs = 25, Val/Met CONs = 36, Met/Met CONs = 15, Val/Val PDMs = 20, Val/Met PDMs = 31, Met/Met PDMs = 29. PDMs differed sub-significantly from CONs in *BDNF* Val66Met genotype distributions (*p* = 0.071) and allele frequency (*p* = 0.066); there was a trend of excessive Met allele in PDMs than in CONs (Table 1).

**Table 1:** *BDNF* Val66Met (rs6265) genotype distributions and allele frequency.

	**Genotype (*****n*****, %)**			**χ^2^**	***p***	**Allele frequency**		**χ^2^**	***p***
	**Val/Val**	**Val/Met**	**Met/Met**			**Val allele**	**Met allele**		
PDM (*n* = 80)	20 (35.0%)	31 (38.8%)	29 (36.6%)	5.28	0.071	44.40%	55.60%	3.38	0.066
CON (*n* = 76)	25 (32.9%)	36 (47.4%)	15 (19.7%)			56.60%	43.40%		

*Significant differences were tested using chi-square tests (p < 0.05). PDM, primary dysmenorrhea patients; CON, healthy female controls; BDNF, brain-derived neurotrophic factor; Val/Val, Valine/Valine; Val/Met, Valine/Methionine; Met/Met, Methionine/Methionine*.

Table 2 shows the odds ratios of PDM. Treating Val carriers (Val/Val and Val/Met) as the reference group, the odds ratio for Met/Met was statistically significant [(Met/Met PDMs x Val-carrier CONs)/(Val-carrier PDMs x Met/Met CONs)]. Treating Val/Val or Val/Met individually as the reference group, the odds ratios for Met/Met were also statistically significant. In contrast, when treating Met carriers (combining Met/Met and Val/Met as one group) or Val/Met as case (exposed group) with Val/Val as reference group, the odds ratios for Met carriers or Val/Met were not significant. These results implied that the odds of PDM were at least 2.25 higher in Met/Met homozygous females than in Val-carrier females.

**Table 2:** Associations of different *BDNF* Val66Met genotypes with PDM.

**Reference**	**Case**	**Odds ratio**	**95% CI**	**χ^2^**	***p*-value**
Val/Val	Met/Met	2.42	1.03–5.69	4.142	0.042^*^
Val/Met	Met/Met	2.25	1.02–4.93	4.125	0.042^*^
Val carrier	Met/Met	2.31	1.12–4.78	5.248	0.022^*^
Val/Val	Met carrier	1.47	0.73–2.95	1.183	0.277
Val/Val	Val/Met	1.08	0.50–2.30	0.036	0.849

* *Significant odds ratios were tested using chi-square tests (p < 0.05). PDM, primary dysmenorrhea patients; CI, confidence interval; Val/Val, Valine/Valine; Val/Met, Valine/Methionine; Met/Met, Methionine/Methionine; Val carrier, Val/Val and Val/Met; Met carrier, Met/Met and Val/Met*.

### Demographic, pain experiences, and psychological characteristics

For demographic data and menstrual features, no significant main effects of group and genotype, and no interactions of group and genotype were found (Table S2). Thus, *post-hoc* pairwise comparisons were not performed on demographic information. Among the three genotypes in PDMs, there were overall no differences in their menstrual pain experiences except the menstrual pain history (*p* = 0.006; Table S3), though *post-hoc* pairwise comparison revealed no significant difference in menstrual pain history between genotypes.

For psychological characteristics, there were consistently significant main effects of group but no main effects of genotype and no group × genotype interactions. PDMs reported significantly lower quality of life and higher personal emotional adjustment problems than those in CONs (Table S4). PDMs also scored higher in negative mood (depression and anxiety) and negative cognitive style to pain (pain catastrophizing) compared to CONs (Table 3).

**Table 3:** Results of negative mood (anxiety and depression) and negative cognitive style to pain (pain catastrophizing) stratified by group and *BDNF* Val66Met genotype.

	**PDM (*n* = 80)**	**CON (*n* = 76)**	**Group main effect (*F*)**	**Genotype main effect (*F*)**	**Group x Genotype interaction (*F*)**	**Between-group (*p*)**
**ANXIETY**						
BAI (0–63)						
Val/Val	7.25 (7.3)	3.04 (3.0)	*F*_(1, 150)_ = 17.23^**^	*F*_(2, 150)_ = 0.85	*F*_(2, 150)_ = 0.29	0.082
Val/Met	5.94 (5.6)	2.94 (2.5)				0.008^*^
Met/Met	7.07 (5.6)	4.27 (3.7)				0.101
Genotype (*p*)	0.659	0.511				
STAI total score (40–160)						
Val/Val	82.16 (17.3)	66.16 (9.90)	*F*_(1, 143)_ = 23.14^**^	*F*_(2, 143)_ = 1.37	*F*_(2, 143)_ = 0.74	< 0.0005^*^
Val/Met	81.23 (16.0)	71.62 (12.6)				0.012
Met/Met	84.31 (15.8)	74.60 (14.6)				0.049
Genotype (*p*)	0.732	0.123				
State anxiety (20–80)						
Val/Val	37.55 (9.1)	30.88 (5.8)	*F*_(1, 149)_ = 13.27^**^	*F*_(2, 149)_ = 1.43	*F*_(2, 149)_ = 0.36	0.003^*^
Val/Met	37.65 (8.5)	33.08 (9.1)				0.063
Met/Met	39.14 (8.2)	35.33 (7.7)				0.164
Genotype (*p*)	0.571	0.199				
Trait anxiety (20–80)						
Val/Val	45.05 (8.7)	35.28 (5.1)	*F*_(1, 143)_ = 27.82^**^	*F*_(2, 143)_ = 0.57	*F*_(2, 143)_ = 1.08	< 0.0005^*^
Val/Met	43.43 (8.7)	37.50 (6.8)				0.003^*^
Met/Met	44.50 (9.2)	39.27 (7.9)				0.056
Genotype (*p*)	0.772	0.244				
**DEPRESSION**						
BDI (0–63)						
Val/Val	7.80 (8.1)	3.52 (3.4)	*F*_(1, 150)_ = 4.65	*F*_(2, 150)_ = 2.12	*F*_(2, 150)_ = 1.40	0.078
Val/Met	5.48 (5.6)	3.56 (5.3)				0.044
Met/Met	6.93 (6.3)	6.87 (6.2)				0.950
Genotype (*p*)	0.614	0.084				
**PAIN CATASTROPHIZING**						
PCS total score (0–52)						
Val/Val	16.80 (9.1)	4.88 (6.8)	*F*_(1, 146)_ = 44.47^**^	*F*_(2, 146)_ = 0.65	*F*_(2, 146)_ = 0.50	< 0.0005^*^
Val/Met	16.94 (13.3)	8.03 (9.0)				0.003^*^
Met/Met	19.04 (9.4)	5.20 (7.8)				< 0.0005^*^
Genotype (*p*)	0.570	0.271				
Helplessness (0–16)						
Val/Val	7.30 (4.2)	2.12 (2.8)	*F*_(1, 146)_ = 44.47^**^	*F*_(2, 146)_ = 0.65	*F*_(2, 146)_ = 0.50	< 0.0005^*^
Val/Met	7.87 (6.0)	3.61 (4.6)				0.001^*^
Met/Met	8.46 (4.8)	2.33 (3.8)				< 0.0005^*^
Genotype (*p*)	0.711	0.422				
Magnification (0–24)						
Val/Val	2.65 (2.1)	1.24 (1.9)	*F*_(1, 146)_ = 21.03^**^	*F*_(2, 146)_ = 0.41	*F*_(2, 146)_ = 0.29	0.011
Val/Met	3.10 (3.1)	1.48 (1.8)				0.031
Met/Met	3.39 (2.1)	1.27 (1.9)				0.001^*^
Genotype (*p*)	0.460	0.602				
Rumination (0–12)						
Val/Val	6.85 (4.0)	1.52 (2.6)	*F*_(1, 146)_ = 56.14^**^	*F*_(2, 146)_ = 0.07	*F*_(2, 146)_ = 1.96	< 0.0005^*^
Val/Met	5.97 (4.7)	2.94 (3.3)				0.006^*^
Met/Met	7.18 (3.9)	1.60 (2.4)				< 0.0005^*^
Genotype (*p*)	0.454	0.145				

Significance main effects of group, genotype, and group x genotype interactions were tested using two-way ANOVAs (p < 0.01, two-tailed).

* p < 0.005,

** p < 0.00001.

*Significant between-group within-genotype planned comparisons were tested using Mann-Whitney U-tests (p < 0.01, two-tailed). Significant between-genotype differences in each group (“Genotype”) were tested using Kruskal-Wallis H tests (p < 0.01, two-tailed). Score ranges are bracketed after each item name. Data are presented as mean (SD). PDM, primary dysmenorrhea patients; CON, healthy female controls; Val/Val, Valine/Valine; Val/Met, Valine/Methionine; Met/Met, Methionine/Methionine; STAI, Spielberger state-trait anxiety inventory; BAI, Beck anxiety inventory; BDI, Beck depression inventory; PCS, pain catastrophizing scale*.

### Multiscale sample entropy

**Figure 2:**
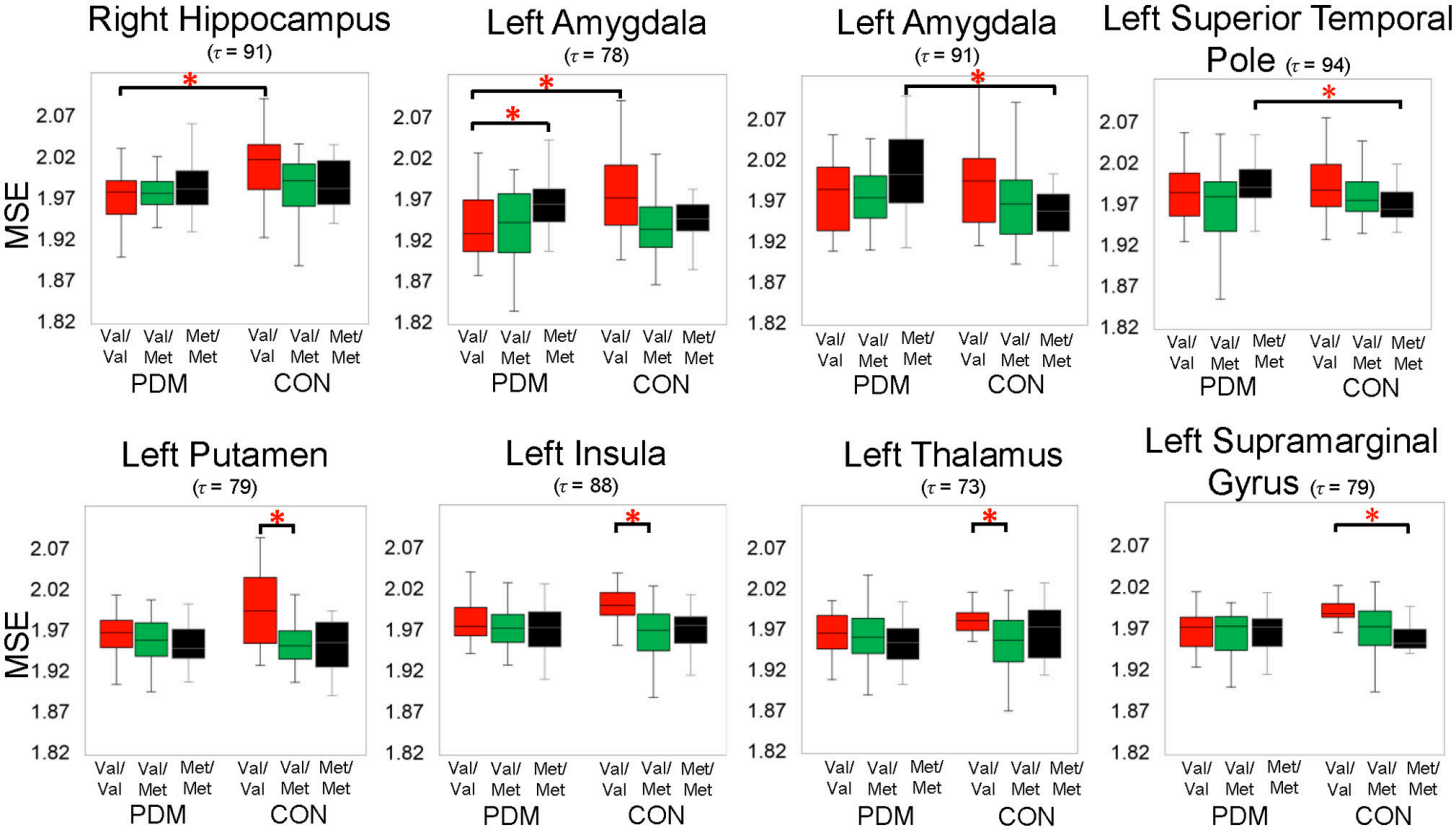
Significant group by genotype interactions on brain complexity in regions, including the left amygdala and right hippocampus. Significant planned comparisons labeled in the figure (^*^) were tested using permutation tests (iterations = 5000, *p* < 0.005, two-tailed). Box plots with 25th (Q1), 50th (median), 75th (Q3) percentiles are demonstrated; the largest and smallest values within 1.5 times the interquartile range (IQR) are plotted as whiskers. Colors: *BDNF* Val/Val (red), Val/Met (green), and Met/Met (black). PDM, primary dysmenorrhea patients; CON, healthy female controls; Val/Val, Valine/Valine; Val/Met, Valine/Methionine; Met/Met, Methionine/Methionine; τ, time scale factors.

In this study, we focused on the interactions of *BDNF* Val66Met polymorphism and long-term menstrual pain. Significant group by genotype interactions were found in brain regions including the hippocampus, amygdala, insula, thalamus, putamen, superior temporal pole, supramarginal gyrus, superior temporal gyrus, and others (Table 4 and Figure 2).

**Table 4:** Significant interactions of *BDNF* Val66Met genotype and group.

**Brain region**	**L/R**	**τ**	**Group x Genotype interaction (*F*)**	**Bonferroni-adjusted (*p*)**
*Limbic regions*				
Hippocampus	R	79,91	3.32, 3.45	0.034, 0.039
Amygdala	L	78,84,88,91	3.62~5.75	0.004~0.029
	R	85	3.97	0.021
Putamen	L	79	3.50	0.033
Superior temporal pole	L	94	3.24	0.042
Middle temporal pole	L	78,98	3.30, 3.95	0.021, 0.040
*Salience network*				
Insula	L	88	3.50	0.033
*Sensorimotor network*				
Thalamus	L	73	3.16	0.045
Supramarginal g	L	79,80	3.48, 3.59	0.030, 0.033
	R	65,66,76,82,88,89	3.07~3.79	0.025~0.049
*Auditory network*				
Superior temporal g	L	92	3.17	0.045
*Visual network*				
Calcarine	L	83	3.30	0.040
Fusiform g	L	87,94	3.21, 3.30	0.040, 0.043
SOG	R	96	3.84	0.024
MOG	L	86,92,98	3.15~3.41	0.026~0.046
IOG	L	74,77,80,87,90,92	3.12~4.97	0.008~0.047

*Significant differences were tested using two-way ANOVAs (Bonferroni-corrected p < 0.05, two-tailed). L, left hemisphere; R, right hemisphere; g, gyrus; SOG, superior occipital gyrus; MOG, middle occipital gyrus; IOG, inferior occipital gyrus*.

**Figure 3:**
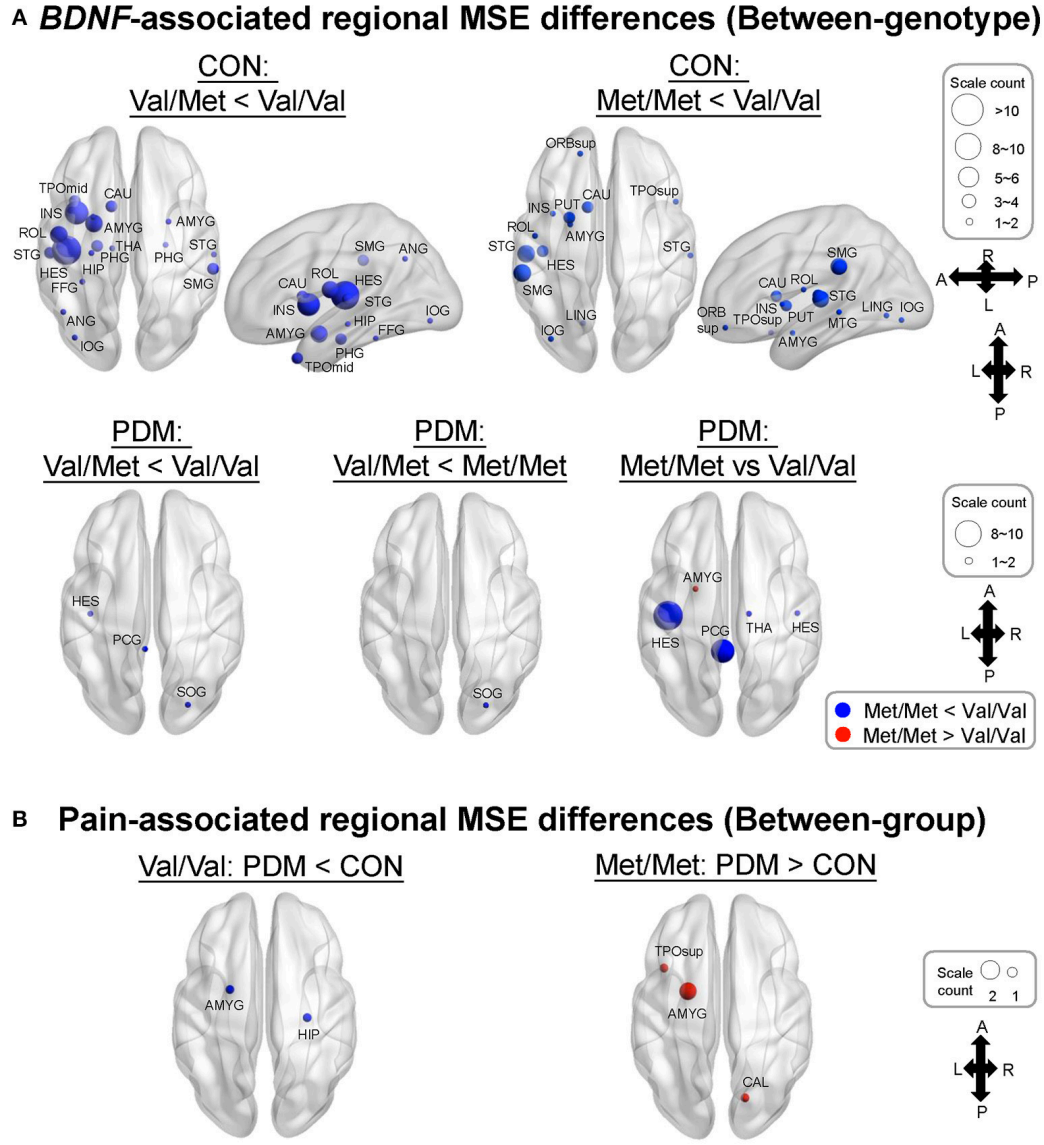
MSE differences between *BDNF* Val66Met genotypes and between groups. **(A)** *BDNF*-associated regional MSE differences in each group (“Between-genotype”). Complexity differences between different genotypes in CONs was greatly diminished in PDMs. Regional MSE values were mostly lower in Val/Met and Met/Met than in Val/Val genotype except in the left amygdala in PDMs. Colors of sphere: Val/Met < Val/Val (blue), Met/Met < Val/Val (blue), Val/Met < Met//Met (blue), Met/Met > Val/Val (red). Size of sphere represents count of scale factors that showed significant MSE differences. **(B)** Pain-associated regional MSE differences in females with the same genotype (“Between-group”). Colors of sphere: PDM < CON (blue), PDM > CON (red). Val/Val, Valine/Valine; Val/Met, Valine/Methionine; Met/Met, Methionine/Methionine.

Specifically, we tested planned comparisons of *BDNF* Val66Met genotypes and groups (Figure 3). For *BDNF*-associated regional MSE differences (between-genotype differences in each group) in CONs (Figure 3A and Table S5), MSE values were all larger in Val/Val CONs than in Val/Met CONs and Met/Met CONs. In PDMs (Figure 3A and Table S6), MSE values were also mostly larger in Val/Val PDMs than in Val/Met PDMs and Met/Met PDMs, except in the left amygdala. Val/Val PDMs manifested significant larger regional MSE in the left posterior cingulate gyrus (PCC) than in Val/Met PDMs and Met/Met PDMs, a phenomenon which was not found in the CON group. We also noticed that in both PDM and CON groups, regional MSE values in the left Heschl's gyrus were all larger in Val/Val than in Val/Met and Met/Met groups.

For pain-associated regional MSE (between-group differences of the same genotype; Figure 3B and Table 5) in Val/Val individuals, MSE values were found to be lower in the right hippocampus and left amygdala in Val/Val PDMs than in Val/Val CONs. On the other hand, in Met/Met individuals, pain-associated regional MSE values were found to be larger in the left amygdala, left superior temporal pole, and right calcarine sulcus in Met/Met PDMs than in Met/Met CONs.

**Table 5:** Pain-associated (between-group) regional MSE differences.

**Brain region**	**L/R**	**Count**	**τ**	***p*-value**	***t*-score**	**Cohen's *d***
**Val/Val: PDM** < **CON (Count** = **2)**						
*Limbic regions*						
Hippocampus^*^	R	1	91	0.0016	−3.41	−1.024
Amygdala^*^	L	1	78	0.0026	−2.98	−0.894
**Met/Met: PDM** > **CON (Count** = **4)**						
*Limbic regions*						
Amygdala	L	2	84,91	0.0007, 0.001	3.34, 3.75	1.063, 1.193
Superior temporal pole	L	1	94	0.0036	3.00	0.953
*Visual network*						
Calcarine s	R	1	83	0.0046	2.83	0.899

Significant planned comparisons tested for whole brain using permutation tests (iterations = 5000, p < 0.005, two-tailed).

* represents significant brain regions that also survived under stricter correction (Bonferroni-adjusted p < 0.05, two-tailed).

*PDM, primary dysmenorrhea patients; CON, healthy female controls; Val/Val, Valine/Valine; Met/Met, Methionine/Methionine; L, left hemisphere; R, right hemisphere; s, sulcus*.

**Figure 4:**
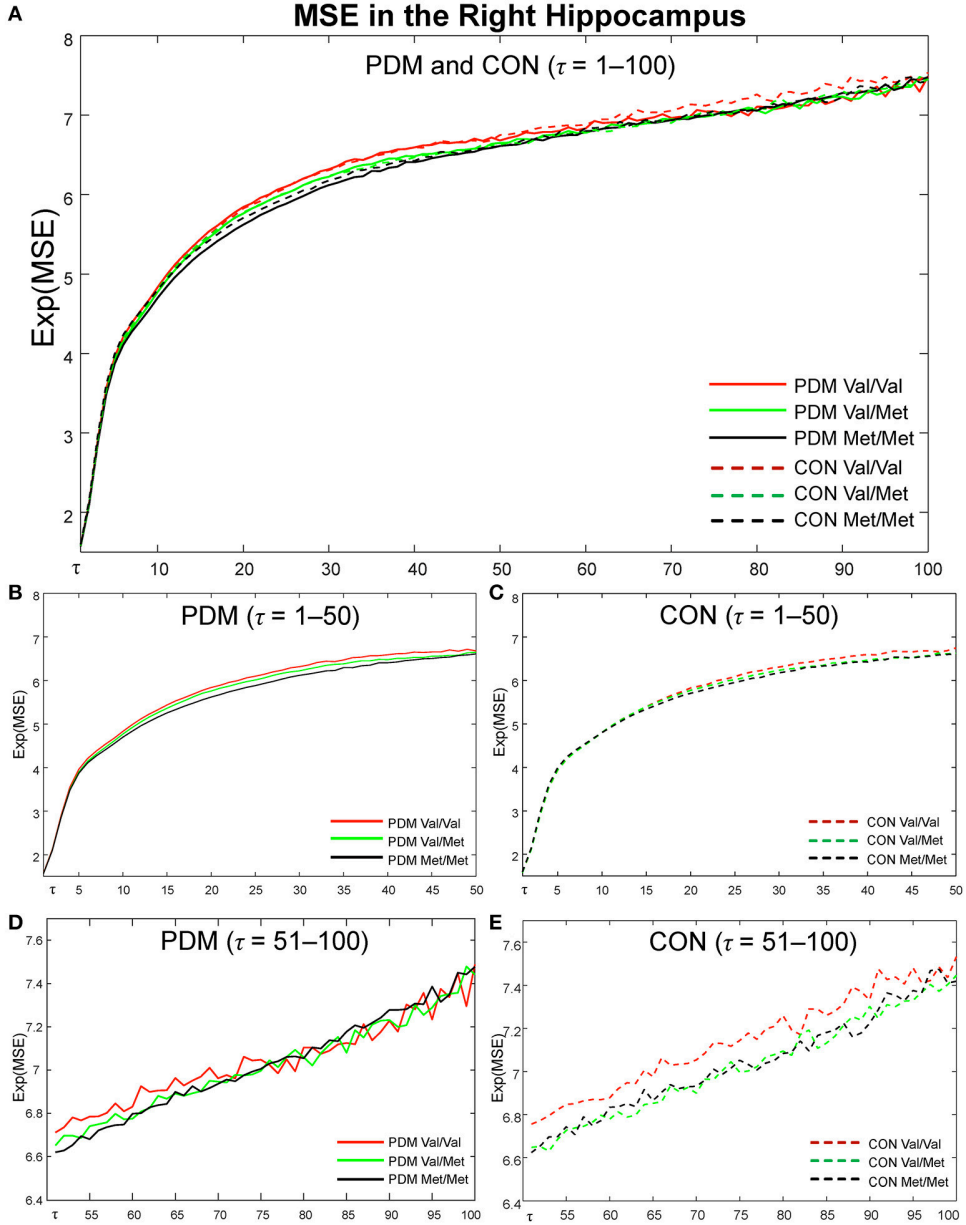
MSE profiles in the right hippocampus. Colors: *BDNF* Val/Val (red), Val/Met (green), Met/Met (black). Lines: PDMs (solid), CONs (dashed). **(A)** MSE profiles of six subgroups from fine to coarse scales across τ = 1 to 100. **(B)** MSE profiles from τ = 1 to 50 in PDM group. **(C)** MSE profiles from τ = 1 to 50 in CON group. **(D)** MSE profiles from τ = 51 to 100 in PDM group. **(E)** MSE profiles from τ = 51 to 100 in CON group. PDM group is illustrated on the left and CON group on the right. Val/Val, Valine/Valine; Val/Met, Valine/Methionine; Met/Met, Methionine/Methionine; τ, time scale factors.

MSE profiles of six subgroups (Val/Val, Val/Met, and Met/Met in PDMs and CONs) in the right hippocampus (Figure 4), one of the most interested regions in our studies, were depicted from time scale factors τ = 1 to 100 for visual comparisons. Overall, Val/Met and Met/Met genotype groups had lower regional MSE than Val/Val group in both PDM and CON groups. At coarse time scales, the differences between Val/Val and Met carriers (Val/Met and Met/Met) were large in CONs, but such differences were diminished in PDMs. Same observations held for those in the left amygdala.

### Correlations between *BDNF*-associated or pain-associated regional MSE and psychological characteristics

**Figure 5:**
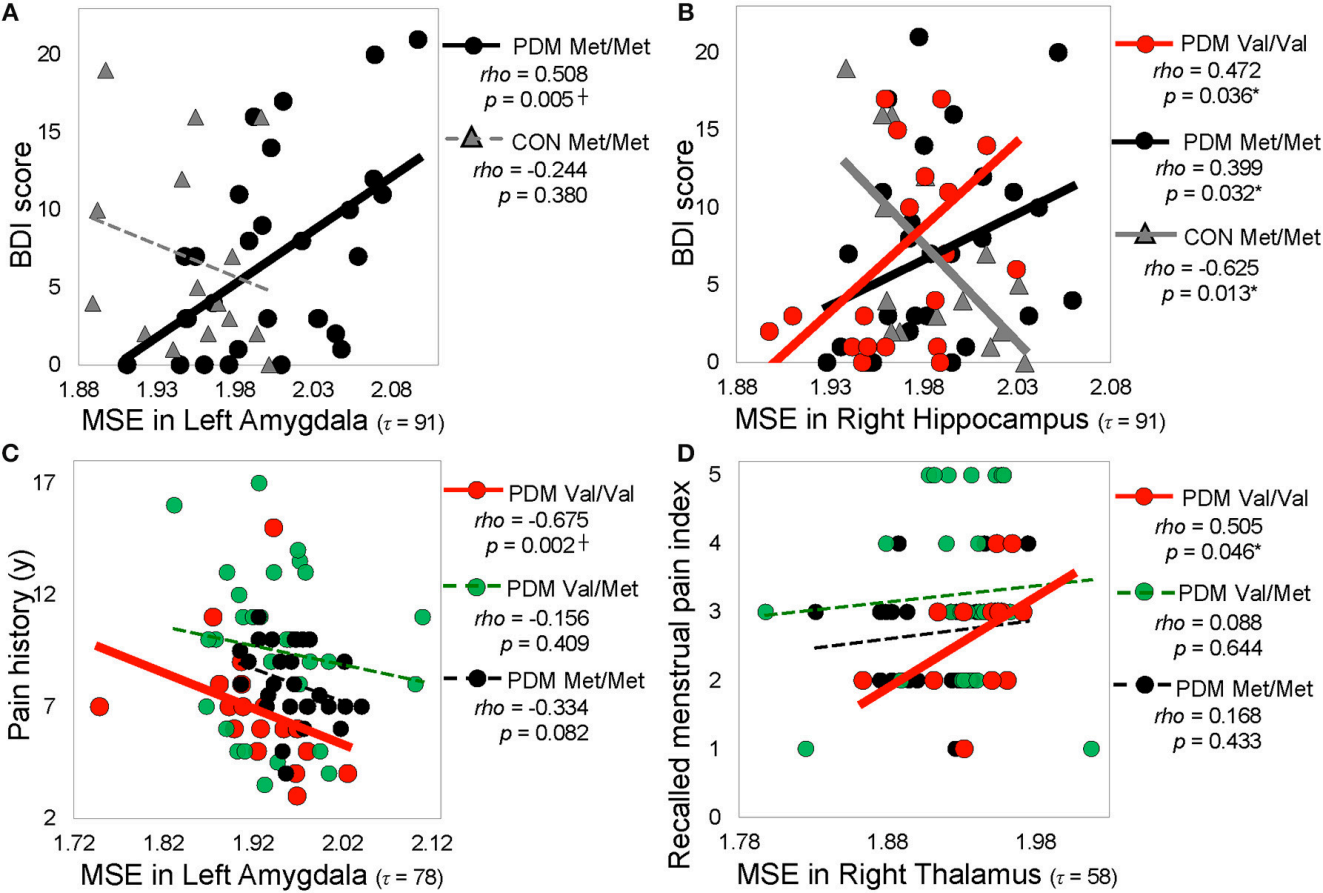
Significant correlations between regional MSE and pain-related psychological characteristics. **(A)** Correlations between MSE in the left amygdala (τ = 91) and depression (BDI score). **(B)** Correlations between MSE in the right hippocampus (τ = 91) and depression score (BDI score). **(C)** Correlations between MSE in the left amygdala (τ = 78) and pain history. **(D)** Correlations between MSE in the right thalamus (τ = = 58) and recalled menstrual pain index. Spearman rho (*p* < 0.05, two-tailed). Significant correlations are plotted as solid lines; correlations that are not significant are plotted as dashed lines. Colors: *BDNF* Val/Val (red), Val/Met (green), and Met/Met (black). Shapes: PDMs (circle), CONs (triangle). MSE, multiscale sample entropy; Val/Val, Valine/Valine; Val/Met, Valine/Methionine; Met/Met, Methionine/Methionine; PDM, primary dysmenorrhea patients; CON, healthy female controls; BDI, Beck depression inventor; McGill pain questionnaire; PPI, present pain index; y, year; τ, time scale factors.

Correlation results were summarized into different resting-state networks and different categories of psychological characteristics (Figure 5 and Table S7). We found that significant correlations in Met/Met group and PDM group mainly emerged in the subcortical regions (such as amygdala, hippocampus) and sensorimotor regions (such as thalamus), whereas significant correlations in Val/Val group and CON group emerged largely in the cortical regions (such as middle temporal gyrus, superior temporal gyrus, fusiform gyrus) and some of the subcortical regions (such as hippocampus). Also, after long-term menstrual pain, the correlations found in CONs were reversed or diminished in PDMs. MSE values in the amygdala showed trends towards negative correlations with both depression and anxiety scores in Met/Met CONs but were positively correlated to those in Met/Met PDMs (Figure 5A). On the other hand, MSE values in the hippocampus were also negatively correlated to depression scores in Met/Met CONs but positively correlated to those in Met/Met PDMs (Figure 5B), and were negatively correlated to anxiety scores in Met/Met CONs but positively correlated to those in Val/Val PDMs.

In PDMs, correlations between pain experiences and regional MSE mainly emerged in the Val/Val group but absent in the Val/Met or Met/Met groups. In Val/Val PDMs, pain chronification (menstrual pain history, PDM onset, menstrual pain duration) experiences were negatively correlated with MSE values. The younger the PDM onset age, or the longer the PDM history/duration, the lower the MSE in the limbic regions including the amygdala (Figure 5C), thalamus, and posterior cingulate gyrus. In contrast, pain intensity experiences (pain score, pain rating indexes) were mostly positively correlated with MSE in the thalamus (Figure 5D) and Heschl's gyrus.

## Discussion

In this study, we used multiscale sample entropy analysis, a powerful tool that quantifies non-linear dynamics in time-varying signals, to investigate whether inter-subject genetic variation interacts with long-term menstrual pain experience to affect brain complexity. First, we found that *BDNF* Val66Met polymorphism (Met/Met homozygosity) is a potential genetic risk factor associated with primary dysmenorrhea, which is in line with previous studies (Lee et al., 2014; Wei et al., 2016b). Second, our findings indicate that long-term menstrual pain experience alters the effects of *BDNF* Val66Met polymorphism on brain complexity. By comparing brain complexity in females of different genotypes with or without menstrual pain, we revealed a characteristic tendency. There was considerable genotype-specific complexity differences in CONs, where Met-carrier (Val/Met and Met/Met) CONs showed extensive lower brain complexity compared to Val/Val CONs. However, the complexity differences were remarkably diminished in PDMs, implying the assaults of chronic recurrent pain on brain complexity. Third, we observed pain-associated brain complexity alterations in the limbic regions, especially the hippocampus and amygdala, in females with same *BDNF* Val66Met genotype.

In our recent study (Low et al., 2017), we categorized female participants according to menstrual pain experience to have a general understanding of brain complexity alterations in PDM. In this study, we further categorized all participants according to *BDNF* Val66Met genotypes together with menstrual pain experience. It is noted that Met allele frequencies of *BDNF* Val66Met polymorphism vary markedly across global populations, ranging from 0 to 72% (Petryshen et al., 2010) with higher frequency in Asian populations (more than 40%) and lower frequency in European populations (around 20%). Hence, the population genetic distribution of *BDNF* Val66Met in Asians allows us to recruit adequate Met-carrier participants to delineate genuine inter-subject genetic variation, as seen in our between-genotype MSE differences. We advise that combining Met carriers (Val/Met and Met/Met) or Val carriers (Val/Met and Val/Val) as one single genotype group, due to the paucity of Met/Met or Val/Val homozygotes, could overlook subtle yet informative genotype-specific changes at the brain level.

Our results demonstrated that the alterations of MSE in PDMs were majorly clustered on large time scales (τ = 50–100), including interactions above scales 73 (Table 4), pain-associated differences above scale 78 (Table 5), *BDNF*-associated differences above scale 50 (Tables S5, S6), and correlations above scale 50 (Table S7). Time scales in MSE are reported to have some correspondences with signal frequencies (Mizuno et al., 2010; Courtiol et al., 2016). Coarse-graining procedure in MSE analysis resembles applying low-pass filtering or down-sampling procedure to the original time-series signal. According to Nyquist-Shannon's sampling theorem, sample entropy value at scale factor τ could reveal the irregularity of the signal under the frequency of (*f*_*s*_/τ)/2 Hz (Courtiol et al., 2016), where *f*_*s*_ is the sampling frequency of the original signal. Given a sampling rate around 1000 Hz in the current study, SE values at time scales 20/50/75/100 might reveal the irregularity of the signal under 25/10/6.7/5 Hz. Our findings of large-scale MSE alterations in PDMs implicate that the resting-state neural complexity altered by the interactions of long-term menstrual pain and *BDNF* Val66Met polymorphism emerged approximately below theta and alpha frequency bands. Moreover, different brain regions exhibited different patterns of alterations, such as the limbic regions (<theta band), the sensorimotor regions (<alpha band), and the default mode network regions (<beta band). These are in line with previous studies, which reported alterations of theta oscillations at limbic regions in PDMs (Lee et al., 2017) and spectral alterations in low frequencies (theta and alpha bands) in chronic pain patients (Pinheiro et al., 2016; Ploner et al., 2017). Thus, MSE could be an important method to explore brain complexity and neural adaptability alterations.

The findings of MSE differences between *BDNF* Val66Met genotypes in healthy female controls support our hypothesis of genotype-specific complexity differences. Regional MSE values in Met-carrier CONs (Val/Met and Met/Met) were extensively lower at different brain regions compared with those in Val/Val individuals (Figure 3A). In healthy conditions, *BDNF* Val/Val homozygotes might serve a protective role on neural complexity, whereas Met allele(s) (Val/Met or Met/Met) might lead to lower neural complexity, implying a defective role of Met allele on the overall brain complexity in healthy females. One common explanation is the “neurotrophic model” that the replacement of Val by Met variant disrupts intracellular trafficking, distribution, and activity-dependent BDNF secretion at synapses in Met carriers (Egan et al., 2003; Chen et al., 2004). As neural complexity might reflect neuronal transients or flexible rapid transitions between neuronal microstates (Friston, 2000; Wang et al., 2018), we reason that Met carriers displayed a general loss of brain complexity compared with Val/Val homozygotes. This result is in line with previous resting-state fMRI study (Wei et al., 2016b) that healthy females with different *BDNF* Val66Met genotypes engaged larger variations in functional connectivity within the descending pain modulatory system. Without the influence of long-term pain experience, healthy individuals might preserve more substantial viability and flexibility in neural dynamics.

However, in PDMs, the between-genotype complexity differences in healthy females was greatly diminished (Figure 3A). This finding suggests that three genotype groups in PDMs demonstrated a less resilient brain system after the experience of long-term menstrual pain. BDNF contributes to the sensitizing capacity of the pain pathways from peripheral nociceptors, spinal level, to brain level, and is cardinally involved in central sensitization of pain (Nijs et al., 2015). Individuals with a single Val allele might “preserve,” at least to some extent, the neural complexity compared to Met carriers. In Val/Val PDMs but not in Met-carrier PDMs, the shorter the PDM history or duration was, the higher the neural complexity was preserved (close to those in Val/Val CONs) in pain-related regions (including the amygdala, thalamus, and posterior cingulate gyrus). These results suggest that the protective role of Val/Val homozygosity with respect to pain chronification is preserved in PDMs and only substitution of both alleles (as in Met/Met PDMs) might lead to loss of complexity in relatively more regions, which might reflect maladaptive neural plasticity upon long-term pain insults.

On the other hand, between-group comparisons of the same genotype (Figure 3B) revealed that neural complexity in the limbic regions (hippocampus, amygdala) was affected by long-term menstrual pain experience in a genotype-specific manner. From our recent study (Low et al., 2017), we learned that the complexity of the hippocampus and amygdala was generally lower in PDMs than in CONs. In the current study, in the hippocampus, Val/Val CONs (the reference group) had the highest regional MSE values and there was no difference among the three genotype subgroups in PDMs. This result suggests that complexity in the hippocampus might be vulnerable to long-term menstrual pain regardless of genotype. Moreover, the hippocampal MSE values were negatively correlated to anxiety and depressive scores in Met/Met CONs but positively correlated in Met/Met PDMs and Val/Val PDMs (Figure 5B), implying possible maladaptive neuroplasticity after the experience of long-term menstrual pain. This speculation is in accordance with the findings in animal study, in which stressful environment leads to increased anxiety-related behaviors in *BDNF* Met/Met mice but not in wild-type mice (Chen et al., 2004). In other words, the effect of chronic pain experience (environmental stress) might preponderantly affect the effect of *BDNF* Val66Met polymorphism on brain complexity.

Interestingly, in the amygdala, Met/Met PDMs showed higher regional MSE values compared with Val/Val PDMs and Met/Met CONs, suggesting that the complexity in the amygdala might be modulated differently by long-term menstrual pain in specific genotype. From the patterns of altered neural complexity observed in the amygdala and hippocampus in PDMs, we speculate the relationships lie between the amygdala, hippocampus, hypothalamus–pituitary–adrenal axis (HPA axis) system, and BDNF. Evidence from cellular to human studies indicates that pain and stress activate the HPA axis. The amygdala and hippocampus may play important yet distinct roles in the HPA axis (Smith and Vale, 2006; Weidenfeld and Ovadia, 2017). BDNF also substantially participates in the regulation of HPA axis activity (Naert et al., 2011). Under stressful environment, hippocampal atrophy and decreased BDNF secretion were found in mice (Chen Z. Y. et al., 2006). In depressive individuals, decreased level of BDNF leads to hippocampal atrophy and prefrontal cortex atrophy (Duman and Monteggia, 2006). Neuronal morphology studies in rats reveal that exposure to chronic stress leads to hippocampus atrophy but amygdala hypertrophy (Vyas et al., 2002). Both acute and chronic stress trigger opposite effects and different temporal profiles on BDNF levels in the amygdala (BLA) vs. hippocampus (CA3) in rats (Lakshminarasimhan and Chattarji, 2012). Thus, chronic stress leads to contrasting patterns of neuronal dendritic remodeling in the hippocampus and amygdala that results in dysregulation of HPA axis (Vyas et al., 2002), which are consistent with our results. Atrophy of the hippocampus causes a loss of hippocampal inhibitory control over the HPA axis, whereas hypertrophy of the amygdala causes a gain in excitatory control over the HPA axis. Our results indicate that the hippocampus might be vulnerable to long-term menstrual pain regardless of *BDNF* Val66Met genotypes, whereas amygdala might be affected by different *BDNF* Val66Met genotypes, implicating a genetic mechanism of variation in brain complexity related to chronic pain. Alternatively, we speculate that the dissociation pattern between the hippocampus and amygdala could be a manifestation of system damping; an adaptive and coping mechanism to relieve the brain from overloaded limbic information while maintaining the pain salience and harmful signal detection. These findings suggest that MSE-brain complexity could be a more sensitive measurement of neurodynamics in comparison to conventional functional connectivity observed in fMRI to reflect the central responses to brain stress (i.e., painful insults).

There is a lack of direct evidence of the association between BDNF or *BDNF* Val66Met polymorphism and neural complexity (as measured by MSE). A clinical case study of a single autism spectrum disorder patient reported electroconvulsive therapy-induced changes of EEG complexity and increased serum BDNF concentrations during and after therapy (Okazaki et al., 2015); yet, the authors did not offer an adequate explanation for the underlying association between BDNF level and EEG complexity. Nevertheless, we speculate a possible link between BDNF and complexity from several points based on the neurobiological functioning of BDNF (Sasi et al., 2017). First, BDNF may be a key mediator and modulator of functional synaptic plasticity, such as activity-induced long-term potentiation and long-term depression (Park and Poo, 2013; Benarroch, 2015). Second, BDNF is also reported to increase the morphology or complexity of dendritic arbors, spines, and microarchitectural integrity, thereby influences structural plasticity (Tolwani et al., 2002; Cohen-Cory et al., 2010; Park and Poo, 2013). Third, both functional and structural plasticity reflect changes in synaptic strength that change in short and long terms. These changes are non-linear and non-stationary and are embedded in time-varying activities of neuronal populations. Therefore, quantifying the irregularity or unpredictability of these activities or synaptic dynamics might shed light on the complexity of the neural system.

From our resting-state regional MSE findings, the effects of *BDNF* Val or Met alleles might be far beyond simple deleterious or protective. We recognized that *BDNF* genetic polymorphism and BDNF protein have complex actions/regulations in the brain that could not be simplified to a single or unifying explanation based on our resting-state brain complexity study at this stage. Other confounding factors, such as age, gender (Stefani et al., 2012), environmental factors, sample size, ethnicity, and phenotype assessment, might also result in controversial findings in *BDNF* genetic studies (Hong et al., 2011; Notaras et al., 2015; Tsai, 2018). Moreover, it is still an ongoing debate whether the Met allele of *BDNF* Val66Met might serve as a deleterious role on brain structures, performances, or health (Autry and Monteggia, 2012; Tingting et al., 2014; Benarroch, 2015; Notaras et al., 2015). Part of these studies was carried out in healthy individuals to avoid possible confounding factors such as illness, medication, or genetic risk factors associated with certain diseases (Harrisberger et al., 2014). For example, a study from healthy Chinese population reported larger gray matter volume in Met/Met homozygotes (Liu et al., 2014), although the underlying mechanism remains elusive. Therefore, for individuals who experience chronic recurrent pain or long-term illnesses, the pain or stress might lead to maladaptive neural plasticity or adaptive coping strategy in different *BDNF* Val66Met carriers due to the activity-dependent manner of *BDNF*, and might come to different conclusions.

Associating genetic variation and chronic recurrent pain with brain complexity may assist in the understanding of individual neural resilience/susceptibility to pain chronification. Moreover, from the polygenic etiology view, it is implausible that *BDNF* is the single gene mediating menstrual pain while gene-gene interactions and epigenetic modulations have their profound contributions on chronic pain development (Denk and McMahon, 2012; Bai et al., 2017). We speculate that epigenetic modulations of long-term menstrual pain on *BDNF* genotypes might better explain our findings. However, to investigate the epigenetic modulations of genetic polymorphism, relevant technologies and tools are required (Weinhold, 2006), and further investigations are needed to fully comprehend the contribution of epigenetic processes to chronic pain states.

There are several limitations in this brain complexity genetic study. First, we only focused on *BDNF* Val66Met genetic polymorphism; there was no information of *BDNF* gene expression, BDNF protein levels, or cortisol levels to test for their associations with brain complexity. Second, other pain-related genetic polymorphisms, such as *BDNF* rs2049046 and G-712A reported in migraine studies (Azimova et al., 2013; Sutherland et al., 2014) or *OPRM1* A118G reported in PDM study (Wei et al., 2017), might also be potential candidates of genetic modulators of chronic pain-sculpted brain complexity. Finally, most neuroimaging genetic studies encounter the problem of small sample sizes compared to traditional genetic studies. Our PDM study samples were particularly limited by challenges of data acquisition of different neuroimaging modalities on the same day, rigorous inclusion/exclusion criteria, and a high exclusion rate. Nevertheless, 156 participants (80 PDMs and 76 CONs) were recruited in the present study, which was relatively large in neuroimaging studies. Future studies are invited to test the neuroimaging genetic results in brain complexity.

## Conclusions

Applying MSE analysis to time-varying MEG signals provides valuable information about neural complexity. We found that PDMs exhibited a general loss of brain complexity in pain-related regions and *BDNF* Val66Met polymorphism is involved in the complexity differences in a genotype-specific manner. Overall, the *BDNF* Val/Val homozygosity might serve as a protective role that preserves the brain complexity. Our results suggest that pain experience preponderantly affects the effect of *BDNF* Val66Met polymorphism on brain complexity, particularly those in the limbic circuits (hippocampus and amygdala), implicating gene-environment interaction on brain complexity.

## Author contributions

L-FC and J-CH conceived and designed the experiments. IL and Y-HL performed the experiments. H-TC performed the clinical assessment. M-WL performed the genotyping. P-CK and Y-SC developed the methodology. IL, C-LT, Y-HL, and M-WL analyzed the data. J-CH provided substantial intellectual input for the work. IL, P-CK, Y-SC, L-FC, and J-CH wrote the manuscript. All authors had reviewed the paper. L-FC and J-CH approved the final submission.

### Conflict of interest statement

The authors declare that the research was conducted in the absence of any commercial or financial relationships that could be construed as a potential conflict of interest.

## Footnotes

^1^This statement has been reproduced with permission of the International Association for the Study of Pain® (IASP). The statement may not be reproduced for any other purpose without permission.

## References

[B1] Alemán-Gómez Y. Melie-García L. Valdés-Hernandez P. (2006). IBASPM: Toolbox for automatic parcellation of brain structures in 12th Annual Meeting of the Organization for Human Brain Mapping. Florence.

[B2] Autry A. E. Monteggia L. M. (2012). Brain-derived neurotrophic factor and neuropsychiatric disorders. Pharmacol. Rev. 64, 238–258. 10.1124/pr.111.005108 22407616 PMC3310485

[B3] Azimova Y. Sergeev A. Skorobogatykh K. (2013). BDNF gene polymorphysm RS2049046 in episodic and chronic migraine. J. Headache Pain 14:P16 10.1186/1129-2377-14-S1-P16

[B4] Bai G. Ren K. Dubner R. (2017). Chapter 8 - Epigenetics in chronic pain” in Translating Epigenetics to the Clinic, ed. Beusekom M. V. (Boston, MA: Academic Press), 185–226.

[B5] Bathina S. Das U. N. (2015). Brain-derived neurotrophic factor and its clinical implications. Arch. Med. Sci. 11, 1164–1178. 10.5114/aoms.2015.56342 26788077 PMC4697050

[B6] Beck A. T. Rush A. J. Shaw B. F. Emery G. (1979). Cognitive Therapy of Depression. New York, NY: The Guilford Press.

[B7] Benarroch E. E. (2015). Brain-derived neurotrophic factor - regulation, effects, and potential clinical relevance. Neurology 84, 1693–1704. 10.1212/wnl.0000000000001507 25817841

[B8] Berkley K. J. (2013). Primary dysmenorrhea: an urgent mandate. Pain: Clinical Updates 21, 1–8. Available online at: http://www.iasp-pain.org/PublicationsNews/NewsletterIssue.aspx?ItemNumber=2062

[B9] Binder D. K. Scharfman H. E. (2004). Brain-derived Neurotrophic Factor. Growth Factors 22, 123–131. 10.1080/08977190410001723308 15518235 PMC2504526

[B10] Cai X. Shi X. Zhang X. Zhang A. Zheng M. Fang Y. (2017). The association between brain-derived neurotrophic factor gene polymorphism and migraine: a meta-analysis. J. Headache Pain 18:13. 10.1186/s10194-017-0725-2 28150221 PMC5289130

[B11] Caumo W. Deitos A. Carvalho S. Leite J. Carvalho F. Dussan-Sarria J. A.. (2016). Motor cortex excitability and BDNF levels in chronic musculoskeletal pain according to structural pathology. Front. Hum. Neurosci. 10:357. 10.3389/fnhum.2016.00357 27471458 PMC4946131

[B12] Chang D. Song D. Zhang J. Shang Y. Ge Q. Wang Z. (2018). Caffeine caused a widespread increase of resting brain entropy. Sci. Rep. 8:2700. 10.1038/s41598-018-21008-6 29426918 PMC5807546

[B13] Chen Y. S. Cheng C. Y. Hsieh J. C. Chen L. F. (2006). Maximum contrast beamformer for electromagnetic mapping of brain activity. IEEE Trans. Biomed. Eng. 53, 1765–1774. 10.1109/TBME.2006.878115 16941832

[B14] Chen Z. Y. Jing D. Bath K. G. Ieraci A. Khan T. Siao C. J.. (2006). Genetic variant BDNF (Val66Met) polymorphism alters anxiety-related behavior. Science 314, 140–143. 10.1126/science.1129663 17023662 PMC1880880

[B15] Chen Z. Y. Patel P. D. Sant G. Meng C. X. Teng K. K. Hempstead B. L.. (2004). Variant brain-derived neurotrophic factor (BDNF) (Met66) alters the intracellular trafficking and activity-dependent secretion of wild-type BDNF in neurosecretory cells and cortical neurons. J. Neurosci. 24, 4401–4411. 10.1523/JNEUROSCI.0348-04.2004 15128854 PMC6729450

[B16] Cohen J. (1988). Statistical Power Analysis for the Behavioral Sciences. Hillsdale, NJ: L. Erlbaum Associates.

[B17] Cohen-Cory S. Kidane A. H. Shirkey N. J. Marshak S. (2010). Brain-derived neurotrophic factor and the development of structural neuronal connectivity. Dev. Neurobiol. 70, 271–288. 10.1002/dneu.20774 20186709 PMC2893579

[B18] Costa M. Goldberger A. L. Peng C. K. (2002). Multiscale entropy analysis of complex physiologic time series. Phys. Rev. Lett. 89:068102. 10.1103/PhysRevLett.89.068102 12190613

[B19] Costa M. Goldberger A. L. Peng C. K. (2005). Multiscale entropy analysis of biological signals. Phys. Rev. E Stat. Nonlin. Soft Matter. Phys. 71(2 Pt 1):021906. 10.1103/PhysRevE.71.021906 15783351

[B20] Courtiol J. Perdikis D. Petkoski S. Muller V. Huys R. Sleimen-Malkoun R.. (2016). The multiscale entropy: guidelines for use and interpretation in brain signal analysis. J. Neurosci. Methods 273, 175–190. 10.1016/j.jneumeth.2016.09.004 27639660

[B21] Denk F. McMahon S. B. (2012). Chronic pain: emerging evidence for the involvement of epigenetics. Neuron 73, 435–444. 10.1016/j.neuron.2012.01.012 22325197 PMC3996727

[B22] Di Lorenzo C. Di Lorenzo G. Daverio A. Pasqualetti P. Coppola G. Giannoudas I.. (2012). The Val66Met polymorphism of the BDNF gene influences trigeminal pain-related evoked responses. J. Pain 13, 866–873. 10.1016/j.jpain.2012.05.014 22901763

[B23] Duman R. S. Monteggia L. M. (2006). A neurotrophic model for stress-related mood disorders. Biol. Psychiatry 59, 1116–1127. 10.1016/j.biopsych.2006.02.013 16631126

[B24] Egan M. F. Kojima M. Callicott J. H. Goldberg T. E. Kolachana B. S. Bertolino A.. (2003). The BDNF val66met polymorphism affects activity-dependent secretion of BDNF and human memory and hippocampal function. Cell 112, 257–269. 10.1016/S0092-8674(03)00035-7 12553913

[B25] Friston K. Breakspear M. Deco G. (2012). Perception and self-organized instability. Front. Comput. Neurosci. 6:44. 10.3389/fncom.2012.00044 22783185 PMC3390798

[B26] Friston K. J. (2000). The labile brain. II. transients, complexity and selection. Philos. Trans. R. Soc. Lond. B Biol. Sci. 355, 237–252. 10.1098/rstb.2000.0561 10724458 PMC1692732

[B27] Friston K. J. (2001). Brain function, nonlinear coupling, and neuronal transients. Neuroscientist 7, 406–418. 10.1177/107385840100700510 11597100

[B28] Generaal E. Milaneschi Y. Jansen R. Elzinga B. M. Dekker J. Penninx B. W. (2016). The brain-derived neurotrophic factor pathway, life stress, and chronic multi-site musculoskeletal pain. Mol. Pain 12:174480691664678. 10.1177/1744806916646783 27145806 PMC4955993

[B29] Ghasemi A. Zahediasl S. (2012). Normality tests for statistical analysis: a guide for non-statisticians. Int. J. Endocrinol. Metab. 10, 486–489. 10.5812/ijem.3505 23843808 PMC3693611

[B30] Haas L. Portela L. V. Bohmer A. E. Oses J. P. Lara D. R. (2010). Increased plasma levels of brain derived neurotrophic factor (BDNF) in patients with fibromyalgia. Neurochem. Res. 35, 830–834. 10.1007/s11064-010-0129-z 20119637

[B31] Hager B. Yang A. C. Brady R. Meda S. Clementz B. Pearlson G. D.. (2017). Neural complexity as a potential translational biomarker for psychosis. J. Affect. Disord. 216, 89–99. 10.1016/j.jad.2016.10.016 27814962 PMC5406267

[B32] Harrisberger F. Spalek K. Smieskova R. Schmidt A. Coynel D. Milnik A.. (2014). The association of the BDNF Val66Met polymorphism and the hippocampal volumes in healthy humans: a joint meta-analysis of published and new data. Neurosci. Biobehav. Rev. 42, 267–278. 10.1016/j.neubiorev.2014.03.011 24674929

[B33] Heisz J. J. McIntosh A. R. (2013). Applications of EEG neuroimaging data: event-related potentials, spectral power, and multiscale entropy. J. Vis. Exp. 76:50131 10.3791/50131 PMC3729183 23851571

[B34] Hong C. J. Liou Y. J. Tsai S. J. (2011). Effects of BDNF polymorphisms on brain function and behavior in health and disease. Brain Res. Bull. 86, 287–297. 10.1016/j.brainresbull.2011.08.019 21924328

[B35] Iacovides S. Avidon I. Baker F. C. (2015). What we know about primary dysmenorrhea today: a critical review. Hum. Reprod. Update 21, 762–778. 10.1093/humupd/dmv039 26346058

[B36] IASP (2011). Visceral and Other Syndromes of the Trunk Apart From Spinal and Radicular Pain in Classification of Chronic Pain: Descriptions of Chronic Pain Syndromes and Definitions of Pain Terms, 2nd Edn (revised) (Seattle, WA: IASP Press). Available online at: http://www.iasp-pain.org/PublicationsNews/Content.aspx?ItemNumber=1673&navItemNumber=677

[B37] Jia Y. Gu H. Luo Q. (2017). Sample entropy reveals an age-related reduction in the complexity of dynamic brain. Sci. Rep. 7:7990. 10.1038/s41598-017-08565-y 28801672 PMC5554148

[B38] Kuo P. C. Chen Y. T. Chen Y. S. Chen L. F. (2017). Decoding the perception of endogenous pain from resting-state MEG. Neuroimage 144(Pt A), 1–11. 10.1016/j.neuroimage.2016.09.040 27746387

[B39] Lakshminarasimhan H. Chattarji S. (2012). Stress leads to contrasting effects on the levels of brain derived neurotrophic factor in the hippocampus and amygdala. PLoS ONE 7:e30481. 10.1371/journal.pone.0030481 22272355 PMC3260293

[B40] Lebedev A. V. Kaelen M. Lovden M. Nilsson J. Feilding A. Nutt D. J.. (2016). LSD-induced entropic brain activity predicts subsequent personality change. Hum. Brain Mapp. 37, 3203–3213. 10.1002/hbm.23234 27151536 PMC6867426

[B41] Lee L. C. Tu C. H. Chen L. F. Shen H. D. Chao H. T. Lin M. W.. (2014). Association of brain-derived neurotrophic factor gene Val66Met polymorphism with primary dysmenorrhea. PLoS ONE 9:e112766. 10.1371/journal.pone.0112766 25383981 PMC4226574

[B42] Lee P. S. Low I. Chen Y. S. Tu C. H. Chao H. T. Hsieh J. C.. (2017). Encoding of menstrual pain experience with theta oscillations in women with primary dysmenorrhea. Sci. Rep. 7:15977. 10.1038/s41598-017-16039-4 29167518 PMC5700160

[B43] Li Z. Fang Z. Hager N. Rao H. Wang Z. (2016). Hyper-resting brain entropy within chronic smokers and its moderation by Sex. Sci. Rep. 6:29435. 10.1038/srep29435 27377552 PMC4932513

[B44] Lin Y. J. (2000). Chinese Beck Anxiety Inventory. Taipei: Chinese Behavioral Science Corporation.

[B45] Liu J. Liu H. Mu J. Xu Q. Chen T. Dun W.. (2017). Altered white matter microarchitecture in the cingulum bundle in women with primary dysmenorrhea: a tract-based analysis study. Hum. Brain Mapp. 38, 4430–4443. 10.1002/hbm.23670 28590514 PMC6866888

[B46] Liu M. E. Huang C. C. Chen M. H. Yang A. C. Tu P. C. Yeh H. L.. (2014). Effect of the BDNF Val66Met polymorphism on regional gray matter volumes and cognitive function in the Chinese population. Neuromol. Med. 16, 127–136. 10.1007/s12017-013-8265-7 24366608

[B47] Liu P. Liu Y. Wang G. Li R. Wei Y. Fan Y.. (2017). Changes of functional connectivity of the anterior cingulate cortex in women with primary dysmenorrhea. Brain Imaging Behav. 12, 710–717. 10.1007/s11682-017-9730-y 28516336

[B48] Liu P. Yang J. Wang G. Liu Y. Liu X. Jin L.. (2016). Altered regional cortical thickness and subcortical volume in women with primary dysmenorrhoea. Eur. J. Pain 20, 512–520. 10.1002/ejp.753 26223337

[B49] Liu Q. Chen Y. F. Fan S. Z. Abbod M. F. Shieh J. S. (2017). EEG artifacts reduction by multivariate empirical mode decomposition and multiscale entropy for monitoring depth of anaesthesia during surgery. Med. Biol. Eng. Comput. 55, 1435–1450. 10.1007/s11517-016-1598-2 27995430

[B50] Low I. Kuo P.-C. Liu Y.-H. Tsai C.-L. Chao H.-T. Hsieh J.-C. (2017). Altered brain complexity in women with primary dysmenorrhea: a resting-state magneto-encephalography study using multiscale entropy analysis. Entropy 19:12 10.3390/e19120680

[B51] Ma W. F. Liu Y. C. Chen Y. F. Lane H. Y. Lai T. J. Huang L. C. (2013). Evaluation of psychometric properties of the Chinese Mandarin version State-Trait Anxiety Inventory Y form in Taiwanese outpatients with anxiety disorders. J. Psychiatr. Ment. Health Nurs. 20, 499–507. 10.1111/j.1365-2850.2012.01945.x 22762356

[B52] McDonough I. M. Nashiro K. (2014). Network complexity as a measure of information processing across resting-state networks: evidence from the Human Connectome Project. Front. Hum. Neurosci. 8:409. 10.3389/fnhum.2014.00409 24959130 PMC4051265

[B53] Melzack R. (1975). The McGill pain questionnaire: major properties and scoring methods. Pain 1, 277–299. 1235985 10.1016/0304-3959(75)90044-5

[B54] Melzack R. (1983). Pain Measurement and Assessment. New York, NY: Raven Press.

[B55] Merighi A. Salio C. Ghirri A. Lossi L. Ferrini F. Betelli C.. (2008). BDNF as a pain modulator. Prog. Neurobiol. 85, 297–317. 10.1016/j.pneurobio.2008.04.004 18514997

[B56] Merskey H. Bogduk N. (2002). Classification of Chronic Pain : Descriptions of Chronic Pain Syndromes and Definitions of Pain Terms, 2nd Edn (revised). Seattle, WA: IASP Press.

[B57] Mizuno T. Takahashi T. Cho R. Y. Kikuchi M. Murata T. Takahashi K.. (2010). Assessment of EEG dynamical complexity in Alzheimer's disease using multiscale entropy. Clin. Neurophysiol. 121, 1438–1446. 10.1016/j.clinph.2010.03.025 20400371 PMC2914820

[B58] Mogil J. S. (2012). Sex differences in pain and pain inhibition: multiple explanations of a controversial phenomenon. Nat. Rev. Neurosci. 13:859–866. 10.1038/nrn3360 23165262

[B59] Naert G. Ixart G. Maurice T. Tapia-Arancibia L. Givalois L. (2011). Brain-derived neurotrophic factor and hypothalamic-pituitary-adrenal axis adaptation processes in a depressive-like state induced by chronic restraint stress. Mol. Cell. Neurosci. 46, 55–66. 10.1016/j.mcn.2010.08.006 20708081

[B60] Nakagawa T. T. Jirsa V. K. Spiegler A. McIntosh A. R. Deco G. (2013). Bottom up modeling of the connectome: linking structure and function in the resting brain and their changes in aging. Neuroimage 80, 318–329. 10.1016/j.neuroimage.2013.04.055 23629050

[B61] Nelson M. Dehaene S. Pallier C. Hale J. (2017). Entropy Reduction correlates with temporal lobe activity in The Workshop on Cognitive Modeling & Computational Linguistics (Valencia).

[B62] Nijs J. Meeus M. Versijpt J. Moens M. Bos I. Knaepen K.. (2015). Brain-derived neurotrophic factor as a driving force behind neuroplasticity in neuropathic and central sensitization pain: a new therapeutic target? Expert Opin. Ther. Targets 19, 565–576. 10.1517/14728222.2014.994506 25519921

[B63] Notaras M. Hill R. van den Buuse M. (2015). The BDNF gene Val66Met polymorphism as a modifier of psychiatric disorder susceptibility: progress and controversy. Mol. Psychiatry 20:916. 10.1038/mp.2015.27 25824305

[B64] Okazaki R. Takahashi T. Ueno K. Takahashi K. Ishitobi M. Kikuchi M.. (2015). Changes in EEG complexity with electroconvulsive therapy in a patient with autism spectrum disorders: a multiscale entropy approach. Front. Hum. Neurosci. 9:106. 10.3389/fnhum.2015.00106 25767444 PMC4341548

[B65] Park C. H. Kim J. Namgung E. Lee D. W. Kim G. H. Kim M.. (2017). The BDNF Val66Met polymorphism affects the vulnerability of the brain structural network. Front. Hum. Neurosci. 11:400. 10.3389/fnhum.2017.00400 28824404 PMC5541016

[B66] Park H. Poo M.- M. (2013). Neurotrophin regulation of neural circuit development and function. Nat. Rev. Neurosci. 14, 7–23. 10.1038/nrn3379 23254191

[B67] Pattwell S. S. Bath K. G. Perez-Castro R. Lee F. S. Chao M. V. Ninan I. (2012). The BDNF Val66Met polymorphism impairs synaptic transmission and plasticity in the infralimbic medial prefrontal cortex. J. Neurosci. 32, 2410–2421. 10.1523/JNEUROSCI.5205-11.2012 22396415 PMC3532006

[B68] Petryshen T. L. Sabeti P. C. Aldinger K. A. Fry B. Fan J. B. Schaffner S. F.. (2010). Population genetic study of the brain-derived neurotrophic factor (BDNF) gene. Mol. Psychiatry 15, 810–815. 10.1038/mp.2009.24 19255578 PMC2888876

[B69] Pezawas L. Verchinski B. A. Mattay V. S. Callicott J. H. Kolachana B. S. Straub R. E.. (2004). The brain-derived neurotrophic factor val66met polymorphism and variation in human cortical morphology. J. Neurosci. 24, 10099–10102. 10.1523/JNEUROSCI.2680-04.2004 15537879 PMC6730170

[B70] Pinheiro E. S. de Queiros F. C. Montoya P. Santos C. L. do Nascimento M. A. Ito C. H.. (2016). Electroencephalographic patterns in chronic pain: a systematic review of the literature. PLoS ONE 11:e0149085. 10.1371/journal.pone.0149085 26914356 PMC4767709

[B71] Ploner M. Sorg C. Gross J. (2017). Brain rhythms of pain. Trends Cogn. Sci. 21, 100–110. 10.1016/j.tics.2016.12.001 28025007 PMC5374269

[B72] Reddy S. Y. Rasmussen N. A. Fourie N. H. Berger R. S. Martino A. C. Gill J.. (2014). Sleep quality, BDNF genotype and gene expression in individuals with chronic abdominal pain. BMC Med. Genomics 7:61. 10.1186/s12920-014-0061-1 25358868 PMC4226913

[B73] Richman J. S. Moorman J. R. (2000). Physiological time-series analysis using approximate entropy and sample entropy. Am. J. Physiol. Heart Circ. Physiol. 278, H2039–2049. 10.1152/ajpheart.2000.278.6.H2039 10843903

[B74] Rorden C. Brett M. (2000). Stereotaxic display of brain lesions. Behav. Neurol. 12, 191–200. 10.1155/2000/421719 11568431

[B75] Sasi M. Vignoli B. Canossa M. Blum R. (2017). Neurobiology of local and intercellular BDNF signaling. Pflügers Archi. Eur. J. Physiol. 469, 593–610. 10.1007/s00424-017-1964-4 28280960 PMC5438432

[B76] Sitges C. Bornas X. Llabres J. Noguera M. Montoya P. (2010). Linear and nonlinear analyses of EEG dynamics during non-painful somatosensory processing in chronic pain patients. Int. J. Psychophysiol. 77, 176–183. 10.1016/j.ijpsycho.2010.05.010 20538021

[B77] Smith S. M. Vale W. W. (2006). The role of the hypothalamic-pituitary-adrenal axis in neuroendocrine responses to stress. Dialogues Clin. Neurosci. 8, 383–395. 17290797 10.31887/DCNS.2006.8.4/ssmith PMC3181830

[B78] Song D. Chang D. Zhang J. Peng W. Shang Y. Gao X.. (2018). Reduced brain entropy by repetitive transcranial magnetic stimulation on the left dorsolateral prefrontal cortex in healthy young adults. Brain Imaging Behav. [Epub ahead of print]. 10.1007/s11682-018-9866-4. 29629499

[B79] Stefani L. C. Torres I. L. de Souza I. C. Rozisky J. R. Fregni F. Caumo W. (2012). BDNF as an effect modifier for gender effects on pain thresholds in healthy subjects. Neurosci. Lett. 514, 62–66. 10.1016/j.neulet.2012.02.057 22395087

[B80] Sutherland H. G. Maher B. H. Rodriguez-Acevedo A. J. Haupt L. M. Griffiths L. R. (2014). Investigation of brain-derived neurotrophic factor (BDNF) gene variants in migraine. Headache 54, 1184–1193. 10.1111/head.12351 24708359

[B81] Tapia-Arancibia L. Rage F. Givalois L. Arancibia S. (2004). Physiology of BDNF: focus on hypothalamic function. Front. Neuroendocrinol. 25, 77–107. 10.1016/j.yfrne.2004.04.001 15571756

[B82] Tingting Y. Lijuan W. Weihong K. Jiajun X. Suping L. Jie C. (2014). Brain-derived neurotrophic factor Val66Met polymorphism association with antidepressant efficacy: a systematic review and meta-analysis. Asia-Pacific Psychiatry 6, 241–251. 10.1111/appy.12148 25231750

[B83] Tolwani R. J. Buckmaster P. S. Varma S. Cosgaya J. M. Wu Y. Suri C.. (2002). BDNF overexpression increases dendrite complexity in hippocampal dentate gyrus. Neuroscience 114, 795–805. 10.1016/S0306-4522(02)00301-9 12220579

[B84] Tononi G. Sporns O. Edelman G. M. (1994). A measure for brain complexity: relating functional segregation and integration in the nervous system. Proc. Natl. Acad. Sci. U.S.A. 91, 5033–5037. 10.1073/pnas.91.11.5033 8197179 PMC43925

[B85] Tsai S.-J. (2018). Critical issues in BDNF Val66Met genetic studies of neuropsychiatric disorders. Front. Mol. Neurosci. 11:156. 10.3389/fnmol.2018.00156 29867348 PMC5962780

[B86] Tseng H. M. Lu J. F. Gandek B. (2003). Cultural issues in using the SF-36 Health Survey in Asia: results from Taiwan. Health Qual. Life Outcomes 1:72. 10.1186/1477-7525-1-72 14641915 PMC385291

[B87] Tu C. H. Niddam D. M. Chao H. T. Chen L. F. Chen Y. S. Wu Y. T.. (2010). Brain morphological changes associated with cyclic menstrual pain. Pain 150, 462–468. 10.1016/j.pain.2010.05.026 20705214

[B88] Tu C. H. Niddam D. M. Yeh T. C. Lirng J. F. Cheng C. M. Chou C. C.. (2013). Menstrual pain is associated with rapid structural alterations in the brain. Pain 154, 1718–1724. 10.1016/j.pain.2013.05.022 23693160

[B89] Tzourio-Mazoyer N. Landeau B. Papathanassiou D. Crivello F. Etard O. Delcroix N.. (2002). Automated anatomical labeling of activations in SPM using a macroscopic anatomical parcellation of the MNI MRI single-subject brain. Neuroimage 15, 273–289. 10.1006/nimg.2001.0978 11771995

[B90] Valencia J. Melia U. Vallverd,ú M. Borrat X. Jospin M. Jensen E. (2016). Assessment of nociceptive responsiveness levels during sedation-analgesia by entropy analysis of EEG. Entropy 18:103 10.3390/e18030103

[B91] Vasant D. H. Payton A. Algladi T. Mistry S. Hamdy S. (2011). Early evidence implicating the brain derived neurotrophic factor (BDNF) val66met polymorphism in the pathogenesis of oesophageal visceral sensitivity. Gut 60(Suppl. 1), A166–A166. 10.1136/gut.2011.239301.353

[B92] Vossen H. Kenis G. Rutten B. van Os J. Hermens H. Lousberg R. (2010). The Genetic Influence on the Cortical Processing of Experimental Pain and the Moderating Effect of Pain Status. PLOS ONE 5:e13641. 10.1371/journal.pone.0013641 21049025 PMC2964315

[B93] Vyas A. Mitra R. Shankaranarayana Rao B. S. Chattarji S. (2002). Chronic stress induces contrasting patterns of dendritic remodeling in hippocampal and amygdaloid neurons. J. Neurosci. 22, 6810–6818. 10.1523/JNEUROSCI.22-15-06810.2002 12151561 PMC6758130

[B94] Wang B. Niu Y. Miao L. Cao R. Yan P. Guo H.. (2017). Decreased complexity in Alzheimer's disease: resting-state fMRI evidence of brain entropy mapping. Front. Aging Neurosci. 9:378. 10.3389/fnagi.2017.00378 29209199 PMC5701971

[B95] Wang D. J. J. Jann K. Fan C. Qiao Y. Zang Y.-F. Lu H. (2018). Neurophysiological basis of multi-scale entropy of brain complexity and its relationship with functional connectivity. Front. Neurosci. 12:352 10.3389/fnins.2018.00352 29896081 PMC5986880

[B96] Wang Z. Li Y. Childress A. R. Detre J. A. (2014). Brain entropy mapping using fMRI. PLOS ONE 9:e89948 10.1371/journal.pone.0089948 24657999 PMC3962327

[B97] Wei S. Y. Chao H. T. Tu C. H. Li W. C. Low I. Chuang C. Y.. (2016a). Changes in functional connectivity of pain modulatory systems in women with primary dysmenorrhea. Pain 157, 92–102. 10.1097/j.pain.0000000000000340 26307856

[B98] Wei S. Y. Chao H. T. Tu C. H. Lin M. W. Li W. C. Low I.. (2016b). The BDNF Val66Met polymorphism is associated with the functional connectivity dynamics of pain modulatory systems in primary dysmenorrhea. Sci. Rep. 6:23639. 10.1038/srep23639 27010666 PMC4806293

[B99] Wei S. Y. Chen L. F. Lin M. W. Li W. C. Low I. Yang C. J.. (2017). The OPRM1 A118G polymorphism modulates the descending pain modulatory system for individual pain experience in young women with primary dysmenorrhea. Sci. Rep. 7:39906. 10.1038/srep39906 28057931 PMC5216367

[B100] Weidenfeld J. Ovadia H. (2017). The role of the amygdala in regulating the hypothalamic-pituitary-adrenal axis in The Amygdala: Where Emotions Shape Perception, Learning and Memories, ed Ferry B. (London: IntechOpen), 173–186. 10.5772/67828

[B101] Weinhold B. (2006). Epigenetics: the science of change. Environ. Health Perspect. 114, A160–167. 10.1289/ehp.114-a160 16507447 PMC1392256

[B102] Wilson D. Lipsey M. (2001). Practical Meta-Analysisi Effect Size Calculator [Online]. Available online at: https://www.campbellcollaboration.org/escalc/html/EffectSizeCalculator-SMD2.php

[B103] Wu T. H. Tu C. H. Chao H. T. Li W. C. Low I. Chuang C. Y.. (2016). Dynamic changes of functional pain connectome in women with primary dysmenorrhea. Sci. Rep. 6:24543. 10.1038/srep24543 27089970 PMC4835697

[B104] Wu W.-T. Lin H.-T. Wang J.-D. Kuo C.-C. (1999). Basic Personality Inventory. Taipei: Psychological Publishing Co., Ltd.

[B105] Yang A. C. Tsai S. J. (2013). Is mental illness complex? From behavior to brain. Prog. Neuropsychopharmacol. Biol. Psychiatry 45, 253–257. 10.1016/j.pnpbp.2012.09.015 23089053

[B106] Yang A. C. Wang S. J. Lai K. L. Tsai C. F. Yang C. H. Hwang J. P.. (2013). Cognitive and neuropsychiatric correlates of EEG dynamic complexity in patients with Alzheimer's disease. Prog. Neuropsychopharmacol. Biol. Psychiatry 47, 52–61. 10.1016/j.pnpbp.2013.07.022 23954738

[B107] Yao Y. Lu W. L. Xu B. Li C. B. Lin C. P. Waxman D.. (2013). The increase of the functional entropy of the human brain with age. Sci. Rep. 3:2853. 10.1038/srep02853 24103922 PMC3793229

[B108] Yap J. C. Lau J. Chen P. P. Gin T. Wong T. Chan I.. (2008). Validation of the Chinese pain catastrophizing scale (HK-PCS) in patients with chronic pain. Pain Med. 9, 186–195. 10.1111/j.1526-4637.2007.00307.x 18298701

[B109] Zhou F. Zhuang Y. Gong H. Zhan J. Grossman M. Wang Z. (2016). Resting state brain entropy alterations in relapsing remitting multiple sclerosis. PLoS ONE 11:e0146080. 10.1371/journal.pone.0146080 26727514 PMC4699711

