# Supplementary material for: Interactions of BDNF Val66Met Polymorphism and Menstrual Pain on Brain Complexity
Source: Front Neurosci. 2018 Nov 20;12:826. doi: 10.3389/fnins.2018.00826 (PMC6256283; doi:10.3389/fnins.2018.00826)
Supplement: Supplementary file 1 [file Table_1.docx]

Supplementary Material

Interactions of *BDNF* Val66Met Polymorphism and Menstrual Pain on Brain Complexity

Intan Low^1,2^, Po-Chih Kuo^3^, Cheng-Lin Tsai^4^, Yu-Hsiang Liu^1^, Ming-Wei Lin^5^, Hsiang-Tai Chao^6^, Yong-Sheng Chen^7,8^, Jen-Chuen Hsieh^1,2,9*^, and Li-Fen Chen^1,2,4,9^*

*** Correspondence:** Jen-Chuen Hsieh: [jchsieh@ym.edu.tw](mailto:jchsieh@ym.edu.tw); Li-Fen Chen: [lfchen@ym.edu.tw](mailto:lfchen@ym.edu.tw)

Supplementary Table S1│Hardy–Weinberg equilibrium test.

| *BDNF*-rs6265 | Observed (%) | Expected (%) | *χ*^2^ | *p*-value |
| --- | --- | --- | --- | --- |
| CON (n = 76) | |  |  |  |
| Val/Val | 25 (32.9%) | 24.3 (32.0%) | 0.098 | 0.952 |
| Val/Met | 36 (47.4%) | 37.3 (49.1%) |  |  |
| Met/Met | 15 (19.7%) | 14.3 (18.9%) |  |  |
| PDM (n = 80) |  | | | |
| Val/Val | 20 (25.0%) | 15.8 (19.8%) | 3.700 | 0.157 |
| Val/Met | 31 (38.8%) | 39.5 (49.4%) |  |  |
| Met/Met | 29 (36.3%) | 24.8 (31.0%) |  |  |
| All (n = 156) |  | | | |
| Val/Val | 45 (28.9%) | 39.5 (25.3%) | 3.101 | 0.212 |
| Val/Met | 67 (43.0%) | 78.0 (50.0%) |  |  |
| Met/Met | 44 (28.2%) | 38.5 (24.7%) |  |  |

Significant differences were tested using *chi*-square tests (*p* < 0.05). PDM, primary dysmenorrhea patients; CON, healthy female controls; BDNF, brain-derived neurotrophic factor; Val/Val, Valine/Valine; Val/Met, Valine/Methionine; Met/Met, Methionine/Methionine.

Supplementary Table S2│Results of demographic data stratified by group and *BDNF* Val66Met genotype.

|  | PDM (n=80) | CON (n=80) | Group main effect (*F*) | Genotype main effect (*F*) | Group x Genotype interaction (*F*) |
| --- | --- | --- | --- | --- | --- |
| Age (y/o) |  |  |  |  |  |
| Val/Val | 22.42 (1.8) | 24.59 (2.7) | *F*(1,150)= 4.70 | *F*(2,150)= 1.68 | *F*(2,150)= 4.07 |
| Val/Met | 24.22 (2.6) | 23.93 (2.3) |  |  |  |
| Met/Met | 23.04 (1.8) | 23.59 (1.6) |  |  |  |
| Menarche (y/o) |  |  |  |  |  |
| Val/Val | 12.00 (1.1) | 12.54 (1.3) | *F*(1,149)= 2.81 | *F*(2,149)= 0.47 | *F*(2,149)= 0.33 |
| Val/Met | 11.94 (1.2) | 12.34 (1.3) |  |  |  |
| Met/Met | 12.33 (1.1) | 12.43 (1.5) |  |  |  |
| Menstruating years (y) |  |  |  |  |  |
| Val/Val | 10.08 (1.9) | 11.70 (2.9) | *F*(1,149)= 0.29 | *F*(2,149)= 2.07 | *F*(2,149)= 2.93 |
| Val/Met | 11.94 (3.2) | 11.20 (2.4) |  |  |  |
| Met/Met | 10.36 (2.0) | 11.29 (2.6) |  |  |  |
| Menstrual cycle (d) |  |  |  |  |  |
| Val/Val | 29.92 (1.9) | 29.64 (1.0) | *F*(1,147)= 0.33 | *F*(2,147)= 0.45 | *F*(2,147)= 1.42 |
| Val/Met | 29.80 (1.4) | 29.71 (1.6) |  |  |  |
| Met/Met | 29.05 (2.0) | 29.90 (2.1) |  |  |  |
| Handedness (%) |  |  |  |  |  |
| Val/Val | 81.78 (19.0) | 83.80 (18.1) | *F*(1,148)= 0.03 | *F*(2,148)= 0.34 | *F*(2,148)= 0.05 |
| Val/Met | 81.49 (19.2) | 81.21 (17.9) |  |  |  |
| Met/Met | 79.31 (20.7) | 79.27 (25.7) |  |  |  |

Significant main effects of group and genotype, and group x genotype interactions were tested using two-way ANOVAs (*p* < 0.01, two-tailed). Since no significant group x genotype interaction and main effects of group and genotype were found, *post-hoc* pairwise comparisons were not performed. Units are bracketed after each item name. Data are presented as mean (SD). PDM, primary dysmenorrhea patients; CON, healthy female controls; Val/Val, Valine/Valine; Val/Met, Valine/Methionine; Met/Met, Methionine/Methionine; y/o, years old; y, year, d, day.

Supplementary Table S3│Results of menstrual pain experiences in PDMs stratified by *BDNF* Val66Met genotype.

| Menstrual pain experience | PDM total (n=80) | Val/Val (n=20) | Val/Met (n=31) | Met/Met (n=29) | Genotype effect | |  |
| --- | --- | --- | --- | --- | --- | --- | --- |
|  |  |  |  |  | *χ*^2^ | *p* | |
| Recalled verbal pain score (0-10) | 6.85 (1.5) | 6.55 (1.6) | 6.92 (1.5) | 7.00 (1.3) | 0.892 | 0.640 | |
| MPQ: Recalled PPI (1-5) | 2.96 (1.1) | 2.69 (0.8) | 3.27 (1.2) | 2.78 (1.1) | 3.212 | 0.201 | |
| MPQ: Recalled PRI scores (0-78) | 35.8 (14.4) | 32.3 (14.6) | 37.1 (14.3) | 36.5 (14.5) | 0.872 | 0.647 | |
| Sensory (0-42) | 18.22 (8.2) | 17.28 (8.6) | 18.32 (8.0) | 18.69 (8.3) | 0.354 | 0.838 | |
| Affective (0-14) | 5.19 (3.3) | 3.94 (2.9) | 5.55 (3.4) | 5.59 (3.3) | 3.252 | 0.197 | |
| Evaluative (0-5) | 3.45 (1.9) | 3.50 (1.7) | 3.58 (1.9) | 3.28 (1.9) | 0.326 | 0.850 | |
| Miscellaneous (0-17) | 8.91 (4.1) | 7.56 (4.2) | 9.68 (4.2) | 8.93 (3.8) | 3.239 | 0.198 | |
| Pain history (y) | 8.33 (3.0) | 6.84 (2.7) | 9.52 (3.6) | 8.05 (1.7) | 10.30 | 0.006* | |
| PDM onset (y/o) | 14.56 (2.0) | 14.94 (1.9) | 14.26 (2.0) | 14.63 (1.9) | 1.433 | 0.488 | |
| Absenteeism (%) | 54.7% | 55.0% | 51.9% | 57.1% | 0.156 | 0.925 | |
| No absenteeism (%) | 45.3% | 45.0% | 48.2% | 42.9% |  |  | |
| Medication (%) | 54.8% | 55.6% | 59.3% | 50.0% | 0.481 | 0.786 | |
| No medication (%) | 45.2% | 44.4% | 40.7% | 50.0% |  |  | |

*Significant genotype differences in menstrual pain experiences in PDMs were tested using Kruskal-Wallis *H* tests (*p* < 0.01, two-tailed). *Post-hoc* pairwise comparisons were tested using Dunn-Bonferroni adjusted *p* < 0.01 (two-tailed) and no between-genotype difference was found. Recalled scores are average recalled menstrual pain intensities over the last six months. Unit or score range are bracketed after each item name. Data are presented as mean (SD). PDM, primary dysmenorrhea patients; Val/Val, Valine/Valine; Val/Met, Valine/Methionine; Met/Met, Methionine/Methionine; MPQ, McGill pain questionnaire; PPI, present pain index; PRI, pain rating index; y, year; y/o, years old.

Supplementary Table S4│Results of quality of life and personal emotional adjustment problems stratified by group and *BDNF* Val66Met genotype.

|  | PDM (n=80) | CON (n=76) | Group main effect (*F*) | Genotype main effect (*F*) | Group x Genotype interaction (*F*) | Between-group (*p*) |
| --- | --- | --- | --- | --- | --- | --- |
| Quality of life |  |  |  |  |  |  |
| SF36 total score (0-200) | |  |  |  |  |  |
| Val/Val | 95.71 (12.0) | 112.31 (5.6) | *F*(1,145)= 52.46** | *F*(2,145)= 1.55 | *F*(1,145)= 0.78 | < 0.0005* |
| Val/Met | 92.42 (15.1) | 108.49 (8.1) |  |  |  | < 0.0005* |
| Met/Met | 94.59 (14.9) | 105.4 (12.4) |  |  |  | 0.025 |
| Genotype (*p*) | 0.892 | 0.066 |  |  |  |  |
| PCS (0-100) |  |  |  |  |  |  |
| Val/Val | 49.67 (7.1) | 53.64 (4.2) | *F*(1,145)= 24.20** | *F*(2,145)= 0.42 | *F*(1,145)= 2.56 | 0.027 |
| Val/Met | 44.65 (12.6) | 55.76 (4.1) |  |  |  | < 0.0005* |
| Met/Met | 47.37 (11.3) | 53.35 (5.1) |  |  |  | 0.088 |
| Genotype (*p*) | 0.427 | 0.072 |  |  |  |  |
| MCS (0-100) |  |  |  |  |  |  |
| Val/Val | 46.05 (9.7) | 58.67 (5.8) | *F*(1,145)= 24.43** | *F*(2,145)= 1.12 | *F*(1,145)= 2.86 | < 0.0005* |
| Val/Met | 47.77 (9.3) | 52.73 (8.4) |  |  |  | 0.038 |
| Met/Met | 47.22 (10.4) | 52.03 (9.5) |  |  |  | 0.108 |
| Genotype (*p*) | 0.741 | 0.014 |  |  |  |  |
| Personality |  |  |  |  |  |  |
| BPI personal emotional adjustment cluster | | |  |  |  |  |
| Anxiety (0-14) |  |  |  |  |  |  |
| Val/Val | 6.15 (3.0) | 3.36 (2.5) | *F*(1,149)= 14.64* | *F*(2,149)= 0.001 | *F*(2,149)= 2.51 | 0.002* |
| Val/Met | 5.00 (3.3) | 4.54 (2.9) |  |  |  | 0.628 |
| Met/Met | 6.03 (3.2) | 3.47 (3.2) |  |  |  | 0.012 |
| Genotype (*p*) | 0.324 | 0.173 |  |  |  |  |
| Depression (0-14) |  |  |  |  |  |  |
| Val/Val | 3.55 (2.8) | 1.24 (1.3) | *F*(1,149)= 14.47* | *F*(2,149)= 0.34 | *F*(2,149)= 1.25 | 0.003* |
| Val/Met | 3.61 (3.0) | 1.86 (2.1) |  |  |  | 0.004* |
| Met/Met | 3.10 (2.7) | 2.47 (2.6) |  |  |  | 0.364 |
| Genotype (*p*) | 0.761 | 0.283 |  |  |  |  |
| Hypochondriasis (0-14) | |  |  |  |  |  |
| Val/Val | 5.00 (3.1) | 2.48 (2.0) | *F*(1,149)= 37.73** | *F*(2,149)= 0.22 | *F*(2,149)= 0.07 | 0.005* |
| Val/Met | 5.42 (2.9) | 2.54 (2.1) |  |  |  | < 0.0005* |
| Met/Met | 5.10 (3.7) | 2.20 (1.2) |  |  |  | 0.009* |
| Genotype (*p*) | 0.743 | 0.999 |  |  |  |  |

Significance main effects of group, genotype, and group x genotype interactions were tested using two-way ANOVAs (*p* < 0.01, two-tailed). **p* < 0.005, ***p* < 0.00001. Between-group within-genotype planned comparisons were tested using Mann-Whitney U tests (*p* < 0.01, two-tailed). Significant between-genotype differences in each group (“Genotype”) were tested using Kruskal-Wallis *H* tests (*p* < 0.01, two-tailed). Score ranges are bracketed after each item name. Data are presented as mean (SD). PDM, primary dysmenorrhea patients; CON, healthy female controls; Val/Val, Valine/Valine; Val/Met, Valine/Methionine; Met/Met, Methionine/Methionine; SF-36, Short-form-36 Quality of Life Assessment; PCS, physical component summary; MCS, mental component summary; BPI, basic personality inventory.

Supplementary Table S5│*BDNF*-associated regional MSE differences in CON group.

| Brain region | L/R | Count | *τ* | *p*-value (range) | *t*-score (range) | Cohen's *d* |
| --- | --- | --- | --- | --- | --- | --- |
| **CON: Val/Val > Met/Met (Count = 35)** | | | |  |  |  |
| *Limbic regions* |  |  |  |  |  |  |
| Amygdala* | L | 1 | 88 | 0.0044 | 2.720 | 0.888 |
| Putamen* | L | 4 | 71,76,84,88 | 0.0024~ 0.0044 | 2.940~ 3.154 | 0.960~ 1.030 |
| Caudate | L | 4 | 69,74,86,87 | 0.001~ 0.0048 | 2.925~3.637 | 0.955~ 1.188 |
| Superior temporal pole | R | 1 | 63 | 0.0038 | 2.976 | 0.972 |
| *Sensorimotor network* | |  |  |  |  |  |
| Supramarginal g* | L | 5 | 73,79,80,97,100 | 0.0006~ 0.0048 | 2.959~3.570 | 0.966~ 1.166 |
| Rolandic operculum | L | 1 | 100 | 0.0032 | 3.110 | 1.016 |
| *Salience network* |  |  |  |  |  |  |
| Insula* | L | 1 | 73 | 0.0034 | 3.001 | 0.980 |
| SFG, orbital | L | 2 | 81,82 | 0.0012, 0.0014 | 3.280, 3.330 | 1.071, 1.088 |
| *Default mode network* | |  |  |  |  |  |
| Middle temporal g | L | 2 | 82,87 | 0.0006, 0.0046 | 3.015, 3.802 | 0.985, 1.242 |
| *Auditory network* |  |  |  |  |  |  |
| Heschl's g | L | 4 | 84,85,92,99 | 0.0008~ 0.0042 | 3.098~3.850 | 1.012~ 1.257 |
| Superior temporal g | L | 5 | 81,87,92,94,99 | 0.0009~ 0.0044 | 2.968~3.698 | 0.969~ 1.208 |
|  | R | 2 | 75,86 | 0.002, 0.0026 | 3.106,3.242 | 1.014, 1.059 |
| *Visual network* |  |  |  |  |  |  |
| Inferior occipital g* | L | 2 | 74,77 | 0.0024, 0.0026 | 3.168, 3.691 | 1.035 |
| Lingual g | L | 1 | 76 | 0.0038 | 3.055 | 0.998 |
| **CON: Val/Val > Val/Met (Count = 61)** | | | |  |  |  |
| *Limbic network* |  |  |  |  |  |  |
| Hippocampus* | L | 2 | 71,80 | 0.0032, 0.004 | 2.956, 3.125 | 0.770, 0.814 |
| Putamen* | L | 2 | 79,88 | 0.0002, 0.0038 | 3.078, 4.183 | 0.801, 1.089 |
| Middle temporal pole* | L | 3 | 63,74,90 | 0.0006~ 0.0026 | 3.204~3.599 | 0.834~ 0.937 |
| Amygdala | L | 6 | 57,59,65,69,70,90 | 0.0006~ 0.004 | 3.004~3.474 | 0.782~ 0.904 |
|  | R | 1 | 76 | 0.002 | 3.369 | 0.877 |
| Parahippocampal g | L | 3 | 68,72,86 | 0.002~ 0.0036 | 3.036~3.190 | 0.790~ 0.831 |
|  | R | 1 | 76 | 0.0030 | 3.055 | 0.795 |
| Caudate | L | 3 | 68,69,90 | 0.0034~ 0.0048 | 2.806~2.853 | 0.731~ 0.743 |
| *Default mode network* | |  |  |  |  |  |
| Angular g | L | 2 | 73,91 | 0.0026, 0.0034 | 2.897, 3.152 | 0.754, 0.821 |
| *Salience network* |  |  |  |  |  |  |
| Insula* | L | 9 | 72,73,79,85,87,88,92,94,99 | 0.0002~ 0.0036 | 2.999~4.024 | 0.781~ 1.048 |
| *Sensorimotor network* | |  |  |  |  |  |
| Thalamus* | L | 1 | 73 | 0.0032 | 3.035 | 0.790 |
| Rolandic operculum | L | 5 | 73,78,83,85,100 | 0.0012~ 0.0048 | 2.903~3.910 | 0.756~ 1.018 |
| Supramarginal g | R | 4 | 70,86,89,98 | 0.0002~ 0.005 | 2.840~3.700 | 0.739~ 0.963 |
| *Auditory network* |  |  |  |  |  |  |
| Heschl's g | L | 12 | 47,58,62,64,68,70,72,81,85,86,90,95 | 0.0002~ 0.005 | 2.918~4.013 | 0.760~ 1.045 |
| Superior temporal g | L | 4 | 75,77,81,96 | 0.0004~ 0.004 | 2.919~3.546 | 0.760~ 0.923 |
|  | R | 1 | 75 | 0.0014 | 3.166 | 0.824 |
| *Visual network* |  |  |  |  |  |  |
| Fusiform g | L | 1 | 77 | 0.0044 | 2.986 | 0.777 |
| Inferior occipital g | L | 1 | 74 | 0.0018 | 3.071 | 0.800 |

Significant planned comparisons tested for whole brain using permutation tests (iterations = 5000, *p* < 0.005, two-tailed). * represents significant brain regions that also survived under stricter correction (Bonferroni-adjusted *p* < 0.05, two-tailed). L, left hemisphere; R, right hemisphere; *τ*, time scale factor; CON, healthy female controls; Val/Val, Valine/Valine; Val/Met, Valine/Methionine; Met/Met, Methionine/Methionine; g, gyrus.

Supplementary Table S6│*BDNF*-associated regional MSE differences in PDM group.

| Brain region | L/R | Count | *τ* | *p* (range) | *t* (range) | Cohen's *d* |
| --- | --- | --- | --- | --- | --- | --- |
| **PDM: Val/Val > Met/Met (Count =21)** | | | |  |  |  |
| *Default mode network* | |  |  |  |  |  |
| Posterior cingulate g | L | 8 | 17,23,24,44,45,50,53,64 | 0.0024~0.0048 | 2.846~3.093 | 0.827~ 0.899 |
| *Sensorimotor network* | |  |  |  |  |  |
| Thalamus | R | 2 | 51,58 | 0.0022, 0.0034 | 3.031, 3.076 | 0.881, 0.894 |
| *Auditory network* |  |  |  |  |  |  |
| Heschl's g | L | 10 | 57,58,60,61,81~84,98,99 | 0.0006~0.004 | 2.823~3.841 | 0.821~ 1.116 |
|  | R | 1 | 50 | 0.0034 | 3.141 | 0.913 |
| **PDM: Val/Val > Val/Met (Count = 3)** | | | |  |  |  |
| *Default mode network* | |  |  |  |  |  |
| Posterior cingulate g | L | 1 | 50 | 0.0030 | 2.619 | 0.751 |
| *Auditory network* |  |  |  |  |  |  |
| Heschl's g | L | 1 | 96 | 0.0020 | 3.276 | 0.940 |
| *Visual network* |  |  |  |  |  |  |
| Superior occipital g* | R | 1 | 96 | 0.0008 | 3.346 | 0.960 |
| **PDM: Val/Val < Met/Met (Count = 1)** | | | |  |  |  |
| *Limbic network* | | |  |  |  |  |
| Amygdala | L | 1 | 78 | 0.0044 | -3.021 | -0.878 |
| **PDM: Val/Met < Met/Met (Count = 1)** | | | |  |  |  |
| *Visual network* | | |  |  |  |  |
| Superior occipital g* | R | 1 | 96 | 0.0026 | -3.016 | -0.779 |

Significant planned comparisons tested for whole brain using permutation tests (iterations = 5000, *p* < 0.005, two-tailed). * represents significant brain regions that also survived under stricter correction (Bonferroni-adjusted *p* < 0.05, two-tailed). L, left hemisphere; R, right hemisphere; *τ*, time scale factor; PDM, primary dysmenorrhea patients; Val/Val, Valine/Valine; Val/Met, Valine/Methionine; Met/Met, Methionine/Methionine; g, gyrus.

Supplementary Table S7│Correlations between *BDNF*-associated or pain-associated regional MSE and anxiety-, depressive-, and pain-related psychological characteristics in each subgroup.

| Brain region | L/R | *τ* | Inventory score / Rating | Val/Val | | Val/Met | | Met/Met | | | | |
| --- | --- | --- | --- | --- | --- | --- | --- | --- | --- | --- | --- | --- |
|  |  |  |  | PDM (*rho*) | CON (*rho*) | PDM (*rho*) | CON (*rho*) | PDM (*rho*) | CON (*rho*) | |  |  |
| *Limbic regions* | |  |  |  |  |  |  |  | |  | |  |
| Amygdala | L | 78 | PDM history (y) | -0.675^┼^ | – | -0.156 | – | -0.334 | – | |  |  |
|  | L | 78 | PDM onset (yo) | 0.589* | – | 0.078 | – | 0.179 | – | |  |  |
|  | L | 69 | BPI Anxiety | -0.075 | 0.114 | 0.242 | -0.408* | 0.146 | -0.676^┼^ | |  |  |
|  | L | 78 | BAI | 0.100 | 0.158 | -0.01 | 0.124 | 0.416* | -0.183 | |  |  |
|  | L | 91 | STAI State anxiety | 0.160 | -0.020 | 0.148 | 0.212 | 0.530^┼^ | 0.041 | |  |  |
|  | L | 91 | STAI Total anxiety | -0.023 | -0.054 | 0.08 | 0.271 | 0.568^┼^ | -0.075 | |  |  |
|  | L | 91 | STAI Trait anxiety | -0.214 | -0.025 | 0.084 | 0.099 | 0.450* | -0.187 | |  |  |
|  | L | 78 | BPI Depression | 0.044 | 0.286 | -0.085 | -0.038 | 0.411* | 0.089 | |  |  |
|  | L | 91 | BDI | 0.057 | 0.106 | 0.372* | -0.006 | 0.508^┼^ | -0.244 | |  |  |
|  | L | 88 | BDI | -0.145 | -0.129 | 0.280 | -0.377* | 0.008 | -0.659^┼^ | |  |  |
| Hippocampus | R | 91 | STAI State anxiety | 0.632* | 0.090 | 0.058 | -0.003 | 0.114 | -0.654* | |  |  |
|  | R | 91 | STAI Total anxiety | 0.595* | -0.013 | -0.036 | 0.050 | 0.013 | -0.784* | |  |  |
|  | R | 91 | BDI | 0.472* | -0.024 | 0.106 | -0.23 | 0.399* | -0.625* | |  |  |
| Parahipp g | L | 86 | STAI Trait anxiety | 0.137 | -0.416* | 0.034 | 0.232 | 0.222 | -0.165 | |  |  |
| Putamen | L | 76 | BPI Hypochondriasis | -0.242 | 0.438* | 0.096 | 0.08 | -0.132 | -0.068 | |  |  |
| Caudate | L | 90 | BPI Hypochondriasis | -0.399 | -0.019 | 0.13 | 0.381* | -0.284 | -0.132 | |  |  |
| *Sensorimotor network* | | |  |  |  |  |  |  | |  | | |
| Thalamus | R | 58 | Recalled MPQ PPI | 0.505* | – | 0.088 | – | 0.168 | – | |  |  |
|  | R | 58 | Menstrual pain duration (d) | -0.568* | – | -0.194 | – | 0.074 | – | |  |  |
|  | R | 58 | PDM history (y) | -0.468* | – | -0.097 | – | 0.017 | – | |  |  |
|  | R | 58 | PDM onset (yo) | 0.481* | – | -0.036 | – | 0.095 | – | |  |  |
| SMG | L | 79 | BDI | -0.085 | -0.066 | 0.423* | -0.096 | 0.249 | -0.521* | |  |  |
|  | R | 98 | STAI Trait anxiety | -0.002 | -0.437* | 0.098 | 0.081 | 0.039 | -0.147 | |  |  |
|  | R | 98 | PCS Rumination | 0.225 | 0.162 | 0.169 | -0.369* | 0.140 | -0.070 | |  |  |
|  | R | 98 | PCS Total | 0.254 | 0.04 | 0.153 | -0.371* | 0.379* | -0.079 | |  |  |
| *Default mode network* | | |  |  |  |  |  |  | |  | | |
| PCC | L | 50 | BPI Anxiety | 0.024 | 0.387 | 0.426* | -0.089 | -0.041 | -0.219 | |  |  |
|  | L | 50 | PDM history (y) | -0.263 | – | -0.456* | – | 0.091 | – | |  |  |
|  | L | 50 | Menstrual pain duration (d) | -0.538* | – | 0.134 | – | 0.120 | – | |  |  |
| MTG | L | 87 | BAI | 0.203 | 0.398* | 0.027 | 0.053 | 0.153 | 0.182 | |  |  |
|  | L | 87 | BDI | 0.273 | 0.416* | 0.364* | -0.108 | 0.122 | -0.469 | |  |  |
|  | L | 87 | BPI Depression | 0.218 | 0.521^┼^ | 0.12 | 0.136 | 0.277 | -0.051 | |  |  |
|  | L | 87 | PCS Rumination | 0.133 | 0.439* | 0.197 | -0.039 | 0.155 | -0.225 | |  |  |
| Angular g | L | 91 | BPI Hypochondriasis | 0.179 | 0.460* | -0.076 | 0.071 | -0.082 | -0.338 | |  |  |
| *Salience network* | |  |  |  |  |  |  |  | |  | |  |
| Insula | L | 73 | BPI Hypochondriasis | 0.396 | 0.439* | 0.136 | 0.125 | -0.005 | -0.113 | |  |  |
| *Auditory network* | |  |  |  |  |  |  |  | |  | |  |
| Heschl's g | L | 96 | BPI Anxiety | 0.450* | -0.257 | 0.147 | -0.018 | -0.084 | -0.06 | |  |  |
|  | L | 85 | PCS Rumination | 0.357 | 0.449* | 0.087 | -0.152 | 0.221 | 0.033 | |  |  |
|  | R | 50 | Recalled menstrual pain score | 0.244 | – | -0.037 | – | -0.436* | – | |  |  |
|  | R | 50 | Recalled MPQ PPI | 0.502* | – | 0.172 | – | -0.200 | – | |  |  |
|  | R | 50 | Recalled MPQ PRI Total | 0.493* | – | -0.100 | – | -0.118 | – | |  |  |
|  | R | 50 | Recalled MPQ PRI Sensory | 0.511* | – | -0.074 | – | 0.026 | – | |  |  |
| STG | R | 75 | BPI Anxiety | -0.035 | 0.215 | 0.325 | -0.337* | -0.095 | -0.208 | |  |  |
|  | R | 86 | BAI | 0.206 | 0.420* | 0.079 | -0.153 | 0.11 | -0.180 | |  |  |
|  | R | 86 | BPI Hypochondriasis | 0.165 | 0.319 | 0.051 | 0.007 | 0.259 | -0.516* | |  |  |
|  | R | 75 | BPI Depression | -0.056 | 0.402* | 0.108 | -0.156 | 0.189 | 0.218 | |  |  |
|  | R | 86 | BDI | 0.441 | 0.511^┼^ | 0.200 | -0.293 | 0.267 | -0.282 | |  |  |
|  | R | 86 | BPI Depression | 0.044 | 0.427* | -0.027 | -0.112 | 0.252 | -0.191 | |  |  |
| *Visual network* | |  |  |  |  |  |  |  | |  | |  |
| Fusiform g | L | 77 | STAI Trait anxiety | -0.157 | -0.425* | 0.056 | 0.026 | 0.225 | -0.266 | |  |  |
|  | L | 77 | STAI Total anxiety | -0.153 | -0.457* | 0.126 | 0.099 | 0.247 | -0.204 | |  |  |
| Calcarine s | R | 83 | PCS Rumination | 0.083 | 0.365 | 0.171 | 0.062 | 0.003 | 0.578* | |  |  |
| IOG | L | 74 | PCS Rumination | 0.162 | 0.191 | 0.1 | -0.089 | -0.152 | 0.582* | |  |  |
| SOG | R | 96 | Recalled MPQ PRI Affective | -0.224 | – | 0.011 | – | -0.439* | – | |  |  |

Spearman *rho, p* < 0.05, two-tailed. RSN, Resting-state network; L, left hemisphere; R, right hemisphere; *τ*, time scale factor; PDM, primary dysmenorrhea patients; CON, healthy female controls; g., gyrus; s, sulcus; Parahipp g, parahippocampal gyrus; SMG, supramarginal gyrus; PCC, posterior cingulate gyrus; MTG, middle temporal gyrus; STG, superior temporal gyrus; IOG, inferior occipital gyrus; SOG, superior occipital gyrus; MPQ, McGill pain questionnaire; PPI, present pain index; PRI, pain rating index; y, year; BPI, basic personality inventory; BAI, Beck anxiety inventory; STAI, Spielberger state-trait anxiety inventory; BDI, Beck depression inventory; PCS, pain catastrophizing scale.
